# Transcriptomic and genetic analysis reveals a Zn2Cys6 transcription factor specifically required for conidiation in submerged cultures of *Thermothelomyces thermophilus*

**DOI:** 10.1128/mbio.03111-24

**Published:** 2024-11-27

**Authors:** Florian Drescher, Yang Li, Jose Manuel Villalobos-Escobedo, Stefan Haefner, Lori B. Huberman, N. Louise Glass

**Affiliations:** 1 The Plant and Microbial Biology Department, The University of California, Berkeley, California, USA; 2 Fine Chemicals and Biocatalysis Research, BASF SE, Ludwigshafen am Rhein, Germany; 3 Plant Pathology and Plant-Microbe Biology Section, School of Integrative Plant Science, Cornell University, Ithaca, New York, USA; Karlsruhe Institute of Technology (KIT), Karlsruhe, Germany

**Keywords:** conidiation, *Thermothelomyces*, fungal biotechnology, asexual development, transcriptional regulation

## Abstract

Filamentous fungi are important producers of enzymes and secondary metabolites. The industrial thermophilic species, *Thermothelomyces thermophilus,* is closely related to the model fungus, *Neurospora crassa*. A critical aspect of the filamentous fungal life cycle is the production of asexual spores (conidia), which are regulated by various stimuli, including nutrient availability. Several species of fungi, including *T. thermophilus*, produce conidia under submerged fermentation conditions, which can be detrimental to product yields. In this study, transcriptional profiling of *T. thermophilus* was used to map changes during asexual development in submerged cultures, which revealed commonalities of regulation between *T. thermophilus* and *N. crassa*. We further identified a transcription factor, *res1*, whose deletion resulted in a complete loss of conidia production under fermentation conditions, but which did not affect conidiation on plates. Under fermentation conditions, the ∆*res1* deletion strain showed increased biomass production relative to the wild-type strain, indicating that the manipulation of *res1* in *T. thermophilus* has the potential to increase productivity in industrial settings. Overexpression of *res1* caused a severe growth defect and early conidia production on both plates and in submerged cultures, indicating *res1* overexpression can bypass regulatory aspects associated with conidiation on plates. Using chromatin-immunoprecipitation sequencing, we identified 35 target genes of Res1, including known conidiation regulators identified in *N. crassa*, revealing common and divergent aspects of asexual reproduction in these two species.

**IMPORTANCE:**

Filamentous fungi, such as *Thermothelomyces thermophilus,* are important industrial species and have been harnessed in the Biotechnology industry for the production of industrially relevant chemicals and proteins. However, under fermentation conditions, some filamentous fungi will undergo a switch from mycelial growth to asexual development. In this study, we use transcriptional profiling of asexual development in *T. thermophilus* and identify a transcription factor that specifically regulates the developmental switch to the production of unwanted asexual propagules under fermentation conditions, thus altering secreted protein production. Mutations in this transcription factor Res1 result in the loss of asexual development in submerged cultures but do not affect asexual sporulation when exposed to air. The identification of stage-specific developmental regulation of asexual spore production and comparative analyses of conidiation in filamentous ascomycete species have the potential to further manipulate these species for industrial advantage.

## INTRODUCTION

Filamentous fungi play a key role in a sustainable economy as they can utilize plant biomass and waste streams to produce high-value proteins and enzymes (1). Over the last decade, *Thermothelomyces thermophilus* (previously *Myceliophthora thermophila* or *Sporotrichum thermophile*) (2), a thermophilic ascomycete fungus, has been studied and engineered for biotechnological exploitation (3–7). *T. thermophilus* naturally produces a variety of thermostable enzymes of interest to the biotechnology industry, including cellulases, xylanases, and laccases (8), and has also been used for heterologous protein production (9). In 2011, a highly mutagenized, industrial production strain (C1) was developed, which produces up to 100 g/L of extracellular protein (10).

One of the core developmental steps in the life cycle of filamentous fungi is the production of asexual spores or conidia, which are important for dispersal and as survival structures; conidia also provide inoculum for industrial cultures. The production of conidia is generally coupled with environmental cues such as CO_2_ concentration, circadian clock signaling, and nutrient status (11–13). The genetics and molecular mechanisms of conidial development are most well characterized in the model filamentous fungi *Neurospora crassa* and *Aspergillus nidulans* (Fig. S1). In *T. thermophilus*, similar to other industrial filamentous fungi, such as *Aspergillus niger*, *Trichoderma reesei,* or *Penicillium chrysogenum,* much remains to be understood about genetic mechanisms associated with asexual development.

The model organism *N. crassa* is a close relative of members of the Thermothelomyces genus (2, 3, 14–16). In *N. crassa,* the genetic regulators for the production of asexual (macro-) conidia have been characterized (17). First, the cAMP phosphodiesterase *aconidiate-2* (ACON-2) regulates the formation of minor constrictions of aerial hyphae starting at the apex of the hyphae (Fig. S1) (18, 19). *aconidiate-3* (ACON-3) (18), and the Zn2Cys6 transcription factor (TF) *fluffy* (FL) control the progression to major constrictions between conidia (20, 21). After conidial maturation, the genes *conidial separation-1* (*csp-1*) (18), encoding a zinc finger TF (22) and *conidial separation-2* (*csp-2*) (18), encoding a grainy head-like TF (23), regulate conidial separation (11).

Conidiation in many filamentous ascomycete fungi, including *Penicillium* sp., *Aspergillus sp.,* and *N. crassa*, has been observed in the wild, on agar plates, and in submerged cultures (11, 24–26). Typically, conidiation in submerged cultures is in response to stress, such as carbon and/or nitrogen limitation. For example, in *A. niger*, sporulation in submerged cultures was induced by either a switch to media lacking a carbon source or nitrogen limitation (25). In *N. crassa*, conidiation in submerged cultures can be induced by nitrogen and carbon starvation and also by heat shock of conidia (21, 26–28). In *T. thermophilus*, conidiation occurs in submerged cultures and fermentation conditions (6, 29).

Engineering *T. thermophilus* for biotechnological uses would benefit from a deeper understanding of the conidiation pathway to either promote or prevent conidiation as required. In this study, we characterized morphological and transcriptional transitions associated with conidiation in submerged cultures of *T. thermophilus*. We identified TFs whose function or expression was associated with conidiation in submerged cultures and deleted some of these TFs to characterize their regulatory role. We identified a TF in *T. thermophilus*, Res1, that was required for conidiation in submerged cultures but that does not regulate conidiation on plates. Deletion of *res1* resulted in increased biomass production in submerged cultures, while over-expression resulted in premature conidiation in both plate and submerged cultures. Expression profiling of strains over-expressing *res1* and chromatin-immunoprecipitation-sequencing (ChIP-Seq) of epitope-tagged Res1 revealed genes and processes associated with conidiation in this industrially relevant fungus. Our findings lay the groundwork to understand the conidiation pathway of *T. thermophilus,* including comparative analyses with other filamentous ascomycete species.

## RESULTS

### Conidiation in submerged cultures of *T. thermophilus* is accompanied by a shift in gene expression

**Fig 1:**
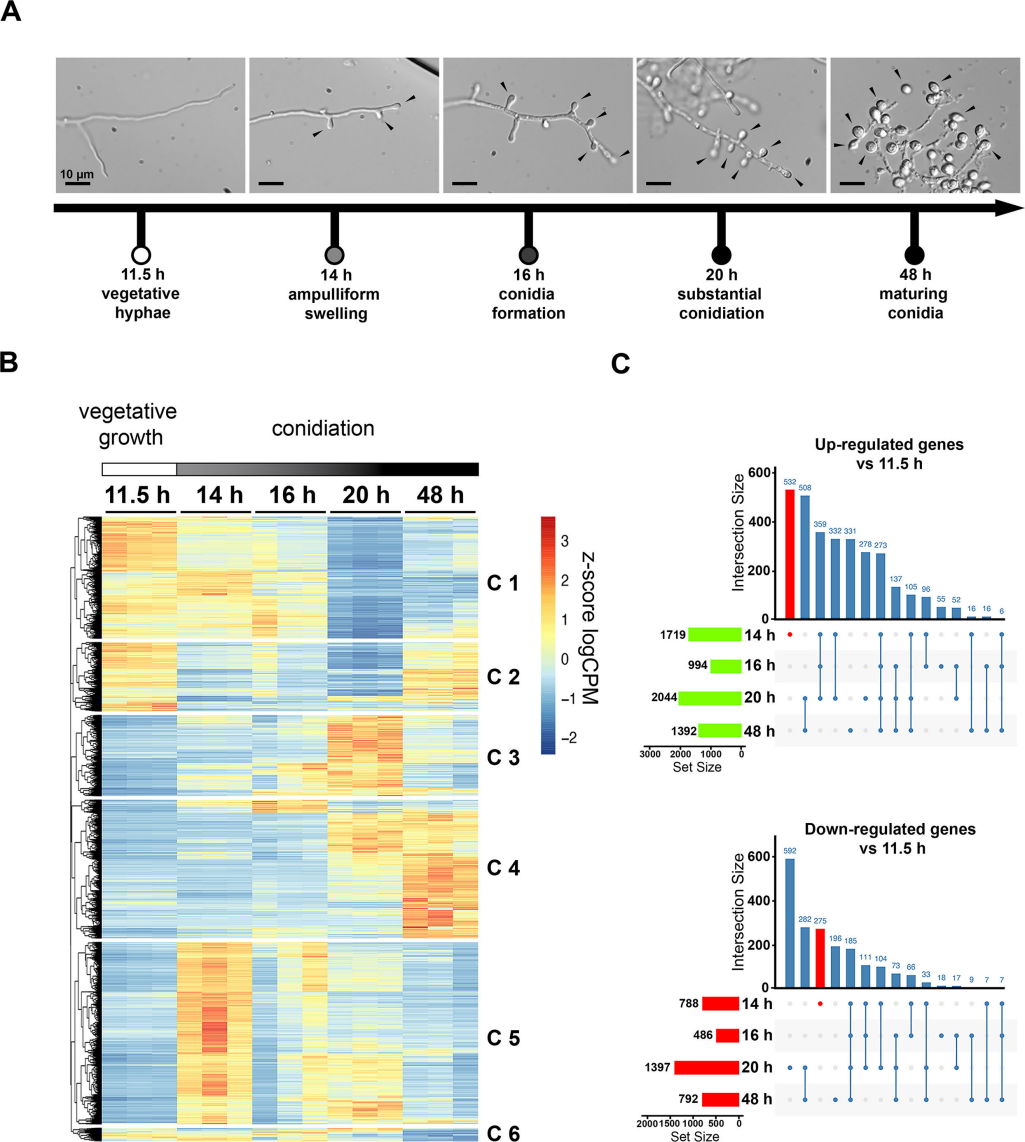
Changes in gene expression patterns during *T. thermophilus* conidiation in submerged culture. (A) Time-course of hyphal and conidial development in submerged cultures in PCM (30). At 11.5 h, only vegetative hyphae were present. At 14 h, hyphal tips showed ampulliform swelling and hyphae showed lateral budding. At 16 h, conidiation had begun at hyphal tips and at lateral buds. At 20 h, conidia formation was more apparent, and at 48 h, conidia were mature with few vegetative hyphae. Black arrows indicate conidiophores and conidia. For a cartoon of conidiation steps, see Fig. S1. (B) Hierarchical clustering of differentially expressed genes during the 48 h time-course. Six distinct clusters of genes with differential expression at one point during the time course were revealed (see Data set S2). The heatmap shows the expression of each triplicate at the different time points normalized as z-score from log counts per million (logCPM). (C) UpSet plot of intersecting upregulated (top) and downregulated (bottom) genes between the different timepoints of the 48 h time-course with the number of differentially regulated genes over the bars. The total number of up- or downregulated genes at the time point is shown as a horizontal bar to the left of the time point.

Early work on *T. thermophilus* conidiation on plates (14) describes the production of single or a few blastoconidia that form by budding from aerial and lateral hyphae (Fig. S1). Although sporulation in submerged cultures/fermentation conditions was reported in *T. thermophilus* (6), a morphological characterization of this process has not been performed. We investigated *T. thermophilus* conidial formation in submerged culture by inoculating *T. thermophilus* conidia into an industrially relevant liquid medium (pre-culture medium, PCM) (30) and observing the development of conidia over time. Only vegetative hyphae were visible after 11.5 h, but at 14 h hyphal tips showed ampulliform swelling at the apex, and lateral budding was visible, indicating the beginning of conidiation (Fig. 1A; Fig. S1). Conidia started to form at hyphal tips at 16 h, and the lateral buds either had constrictions at the base or formed short lateral hyphal branches with a conidium constriction at the tip. At 20 h, most hyphae showed conidial development. Conidia had a drop-like shape with a strong constriction at the base. Short lateral branches had also developed conidia at their tips and in some cases, a second conidium was formed from the hyphal branch. After 48 h in liquid culture, conidia increased in size and started to separate from the hyphae, indicating conidial maturation (Fig. 1A; Fig. S1). Conidial development in submerged cultures of *T. thermophilus* reflected developmental stages associated with sporulation on plates (14).

As with other filamentous fungi, such as *N. crassa* (19), we predicted that gene expression profile changes would occur when hyphae transitioned to asexual development in submerged cultures of *T. thermophilus*. Of the 9,091 genes predicted in the *T. thermophilus* genome, only 2,884 genes (~30%) have been annotated (https://mycocosm.jgi.doe.gov/Spoth2/Spoth2.home.html). Given that *T. thermophilus* is phylogenetically close to *N. crassa*, which has more than 50% of its genome annotated, we used the OrthoFinder program to identify orthologous genes between these two species (31) (Fig. S2) (Materials and Methods) to assign functions to 3,441 genes (an increase of 557 newly annotated genes). EggNOG-mapper v2 (32) was used to increase the number of genes with functional annotations to 5,171 (56%) (Fig. S2; Data set S1) and the EnsemblFungi database (https://fungi.ensembl.org/Thermothelomyces_thermophilus_atcc_42464_gca_000226095/Info/Index) was used to for annotation of a further 138 genes.

To test the hypothesis that differential gene expression occurs during *T. thermophilus* asexual development, we used RNA-Seq to follow gene expression patterns during conidiation in submerged cultures over a 48-h time course. A twofold change in expression level between the 11.5-h time point (prior to conidiation) and any subsequent time point and a false discovery rate (FDR) of <0.05 was used as a threshold for identifying differentially regulated genes; 4,816 genes passed these criteria (Fig. 1B). Differentially regulated genes shared between the different timepoints were visualized using UpSet plots (Fig. 1C). At the 14-h time point, when conidiation begins, 532 genes increased in expression level only at this time point, while 275 genes showed a decrease in expression profile only at this time point, compared to the 11.5-h time point (Fig. 1C, red vertical bar).

Hierarchical clustering revealed six clusters of genes that showed similar expression patterns across the time course (Fig. 1B). We used ShinyGo (33) to identify enriched functional GO categories and pathways in the differentially regulated gene clusters using the *T. thermophilus* genome with a minimum of 5 genes and a maximum of 500 genes per category (size of the GO terms) as a selection threshold. Cluster C1 showed increased expression profiles at 11.5 h, 14 h, and 16 h. GO categories enriched in cluster C1 included cytosolic large ribosomal subunit and protein kinase C binding (8.4-fold enrichment, 2.31e−16 FDR), proteasome (6.5-fold enrichment, 4.02e−06 FDR), and translation related (6.3-fold enrichment, 9.01e−34 FDR) (Fig. 1B; Data set S3). Cluster C2 showed increased expression profiles at 11.5 h and 48 h and showed enrichment in mitochondrial and respiratory-related categories (15.1-fold enrichment, 3.1e−06 FDR) as well as mitochondrial ribosomal categories (8.6-fold enrichment, 2.3e−13 FDR) (Fig. 1B; Data set S3). In cluster C3, no enrichment reached an FDR < 0.05. Genes in cluster C4 showed increased expression levels at 20 h and 48 h when conidia mature. This cluster had enriched categories including seed, conidium formation, and protein-ribulosamine 3-kinase activity (6.3-fold enrichment, 0.06 FDR), conidium formation, ubiquitin conjugation pathway (6.1-fold enrichment, 0.007 FDR), and plasma membrane proteins (eightfold enrichment, 2.5e−06 FDR) (Data set S3). Genes in cluster C5 increased in expression profile at 14 h, coinciding with the beginning of conidiation and early conidial development. The highest fold enrichment were genes involved in DNA replication (3.3-fold enrichment, 0.014 FDR), amino acid metabolism (1.8-fold enrichment, 5.1e−05 FDR), and vesicle transport (1.7-fold enrichment, 0.03 FDR) (Data set S3). Genes within cluster C6 have decreased expression levels at 48 h when conidia mature, and few vegetative hyphae are left. The highest enriched categories were fatty acid synthesis-related (34.4-fold enrichment, 0.018 FDR), cell cycle-related (23-fold enrichment, 0.018 FDR), and organic acid metabolism (2.94-fold enrichment, 0.018 FDR) (Data set S3).

We identified the 25 most highly expressed genes associated with the initiation of conidiation (Data set S2; 14 h_vs_11.5 h). Six of these 25 genes have homologs in other species that are associated with secondary metabolite production (MYCTH_2309486, MYCTH_73236, MYCTH_78013, MYCTH_114667, MYCTH_2303048, and MYCTH_114666). A correlation between secondary metabolite production and conidiation was identified in a number of filamentous fungal species (34). Additional genes included MYCTH_52496, a *CIS3* homolog of *Saccharomyces cerevisiae* which is a mannose-containing glycoprotein in the cell wall involved in cell wall stability (35, 36), and MYCTH_61507, a predicted ABC multidrug transporter. Both MYCTH_52496 and MYCTH_61507 are targets of Res1 (see below).

### *T. thermophilus* homologs of *Aspergillus* and *Neurospora* conidiation-associated genes change expression during growth in submerged cultures

**Fig 2:**
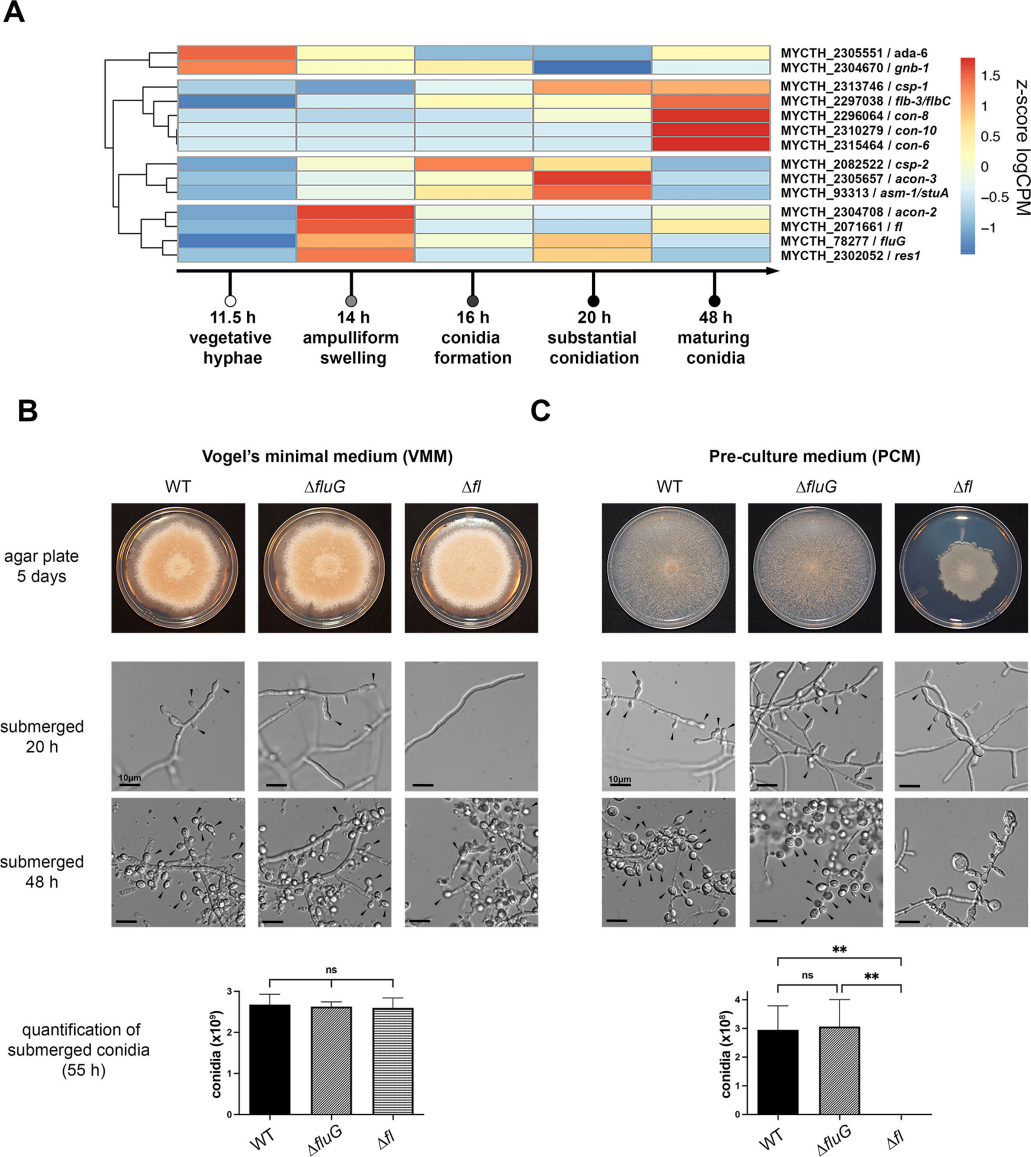
Expression patterns of conserved conidiation regulators showing that the *T. thermophilus fl* homolog regulates conidiation in submerged culture in an environmental condition-dependent fashion. (A) Heatmap of hierarchically clustered, differentially expressed *T. thermophilus* homologs of conidiation-related genes during the 48-h time course. Heatmap shows z-score from log10 counts per million (logCPM). (B) Representative agar plates of *T. thermophilus* WT, *∆fluG,* and *∆fl* mutants after 5 days of growth on plates and the corresponding submerged culture phenotype in Vogel’s minimal medium (VMM) (39) and pre-culture medium (PCM) (30) after 20 and 48 h. Conidial quantification from submerged cultures of WT and the mutants from three biological replicates are shown below. Scale bar 10 µm. Conidia/conidiophores are indicated by black arrows. One-way ANOVA with Tukey’s post-test, ns = non-significant, **P < 0.01.

To identify genes associated with regulating conidiation, we focused our RNA-seq analysis on homologs of genes encoding regulators of conidiation in other fungal species. A survey of homologs of conidiation genes from *N. crassa* and *Aspergillus sp*. revealed that homologs of 13 *N. crassa* genes were identified in the genome of *T. thermophilus;* two of these *N. crassa* genes also regulated conidiation in *A. nidulans* (*flbC* and *stuA*) (Table S1). One unique *A. nidulans* conidiation regulator homolog was identified in the *T. thermophilus* genome (*fluG*). Of these fourteen conserved genes, 12 *T. thermophilus* homologs showed increased expression levels at certain time points in submerged cultures (Fig. 2A). The *T. thermophilus ada-6* homolog (MYCTH_2305551; Table S1) and the *gnb-1* homolog (MYCTH_2304670; Table S1) both showed increased expression levels at the 11.5-h time point relative to other time points before conidiation was apparent (Fig. 2A). In *N. crassa*, *ada-6* is a global regulator of sexual development and conidiation and positively regulates *fl* and *acon-3*, as well as the conidiation-specific genes *eas*, *con-6*, *con-8*, *con-10*, *con-13*, *pcp-1*, and NCU09357, which are involved in late conidial development (37). Deletion of *ada-6* reduces conidia production in *N. crassa* by more than 20-fold (37). The *gnb-1* locus encodes a heterotrimeric G-protein subunit (GNB-1) that forms a dimer with the G-protein subunit GNG-1 in *N. crassa*. A *gnb-1* deletion is associated with female sterility and induces conidiation in submerged culture (38).

The *T. thermophilus* homologs of the *N. crassa* conidiation-related genes *con-6, con-8,* and *con-10* genes showed the highest expression levels at 48 h, at the end of the conidiation process, although the *con-8* homolog was also expressed at 20 h (Fig. 2A). This expression pattern mirrors that observed for the *con* genes during *N. crassa* conidiation (40, 41). The *N. crassa con-6, con-8,* and *con-10* genes (MYCTH_2315464, MYCTH_2296064, and MYCTH_2310279; Table S1) encode small polypeptides of unknown biochemical function (40–42). Two homologs to genes involved in conidial separation in *N. crassa*, *csp-1,* and *csp-2* also showed increased expression levels during conidiation in submerged cultures. In *N. crassa,* the *csp-1* gene encodes a zinc finger TF and is a regulator of conidial separation and azole resistance (11, 20, 43). The *T. thermophilus* homolog of *csp-1* (MYCTH_2313746; Table S1) showed increased expression levels at 20 h and 48 h (Fig. 2A). The *csp-2* gene in *N. crassa* encodes a grainy head transcription factor involved in cell wall remodeling during the conidiation process (23). Loss-of-function mutations in *csp-2* are blocked in conidial separation at the double-doublet stage (20, 44). Consistent with its role in conidiation in *T. thermophilus*, the homolog of *csp-2* (MYCTH_2082522; Table S1) showed increased expression at 16 h and 20 h.

The *acon-2* gene in *N. crassa* encodes a cAMP phosphodiesterase that regulates minor constriction ring formation during conidiation (19, 44), while the *acon-3* gene encodes a transcription factor that is required for major constriction ring formation (44). The *T. thermophilus* homolog of *acon-2* (MYCTH_2304708; Table S1) showed the highest level of expression at 14 h (Fig. 2A) while the expression levels for the *acon-3* homolog (MYCTH_2305657; Table S1) peaked at 20 h (Fig. 2A). The *N. crassa* conidiation regulator *fl* is highly conserved in *T. thermophilus* (MYCTH_2071661; Table S1); however, its expression level did not meet our threshold for differential gene regulation. Nevertheless, *fl* was upregulated in two of three replicates at 14 h as compared to 11.5 h (Data set S4).

In *Aspergillus species,* conidiation involves a more specialized developmental process, consisting of a foot cell from which one or several stalk cells emerge (Fig. S1). Stalk cells form a multinucleate cell at their tip on which sterigmata, metulae, and phialides form. Phialides divide asymmetrically after mitosis to form long chains of conidia (13, 34, 45). Deletion of the transcription factor *stuA* (*asm-1* in *N. crassa*) prevents stalk cell formation and results in the absence of deformed phialides (46). The *T. thermophilus stuA/asm-1* homolog (MYCTH_93313; Table S1) showed the highest level of expression at 20 h, with significant expression also observed at 16 h.

A major upstream regulator of conidiophore development in *A. nidulans* is *fluG* (47), which is responsible for the production of an extracellular sporulation-inducing signal; FluG is a bifunctional enzyme that participates in a pathway involving a γ-glutamylated intermediate (48). A deletion of *fluG* leads to a fluffy colony lacking conidia. Downstream of *fluG,* the transcriptional activator *flbC* (*flb-3* in *N. crassa*; Table S1) is a positive regulator of the transcription factor *brlA* (no identifiable homolog in *T. thermophilus*), which is a regulator of the switch from vegetative to asexual development (49, 50). Mutations in *flbC/flb-3* in *A. nidulans* and *N. crassa* result in a fluffy phenotype with few or no conidia (51, 52). In *T. thermophilus,* the *fluG* homolog (MYCTH_78277; Table S1) showed the highest expression levels at 14 h followed by 20 h, coinciding with the beginning stages of conidiation (Fig. 2A). The *flbC/flb-3* homolog (MYCTH_2297038; Table S1) showed a slight upregulation at 16 and 20 h but had the highest expression levels at 48 h when conidia are mature (Fig. 2A).

We hypothesized that genes that regulate conidiation in *A. nidulans* and *N. crassa* would also regulate conidiation in *T. thermophilus*. To test this hypothesis, we deleted the *T. thermophilus fluG* homolog (MYCTH_78277) and the *T. thermophilus* homolog of *fl* (MYCTH_2071661) (Fig. S3). Unlike the ∆*fluG* phenotype in *Aspergilli* (47) and the ∆*fl* mutant phenotype in *N. crassa* (53), neither aerial hyphae formation nor conidia production was affected in the *T. thermophilus* ∆*fluG* or ∆*fl* mutants on agar VMM or PCM plates (30, 39) (Fig. 2B; Fig. S4), although the *T. thermophilus* ∆*fl* mutant showed sparser growth on PCM. When the *T. thermophilus* ∆*fluG* and ∆*fl* mutants were grown in submerged cultures in VMM, both mutants were indistinguishable from WT and produced numerous conidia (Fig. 2C). In PCM, the submerged *T. thermophilus* WT strain and the ∆*fluG* mutant both formed conidia after 20 h, but the ∆*fl* mutant failed to form any conidia (Fig. 2C). Instead, the *T. thermophilus* ∆*fl* mutant formed multi-nucleate swollen structures with thick cell walls (Fig. 2C; Fig. S4). VMM (39) and PCM (30) differ in the nitrogen source (NH_4_NO_3_ versus [NH_4]2_SO_4_), carbon source (sucrose versus glucose), trace elements, and presence of Na_3_citrate (VMM only), respectively (See Materials and Methods). These data indicated that neither FluG nor FL has a role in conidiation on agar plates in *T. thermophilus*, but FL affected the production of conidia in submerged cultures under certain nutritional conditions.

### A deletion of the Zn2Cys6 transcription factor *res1* prevents conidiation in submerged cultures

As the ∆*fluG* mutant did not phenotypically affect conidiation in *T. thermophilus* and the ∆*fl* mutant conidiation phenotype was dependent upon nutritional conditions, we focused instead on predicted TFs annotated in the genome (264 total predicted TFs from the Cis_BP database (https://cisbp.ccbr.utoronto.ca; (54) (Data set S1); 92 TF genes were highly upregulated (>3-fold change, FDR < 0.5) (Fig. S5) in submerged cultures of *T. thermophilus*. Hierarchical clustering of these 92 TFs showed two clusters (C2 and C5; Fig. S5) that showed increased expression levels at 14 h and contained 9 and 30 TFs, respectively (Table S2). Homologs of TF genes in Clusters 2 and 5 included *N. crassa* genes that regulate conidiation or have a conidiation phenotype when mutated, including *wc-1*, *vib-1, ada-10, vad-5, tah-5, adv-1, ada-1,* NCU00019, NCU03070, *tah-1*, *sah-4*, NCU03417 and NCU06145 (Table S2).

Based on the analysis of TFs that showed increased expression levels at 14 h, we used double-joint PCR and homologous recombination to create deletion mutants and successfully created mutants in a homeobox TF (MYCTH_2296328) and a zinc finger TF (MYCTH_2302052) (Fig. S3A). The MYCTH_2296328 TF has no functional annotation, and the deletion mutant did not show a phenotype under the conditions tested. The homolog of MYCTH_2302052 was described previously as a regulator of the ER stress response in *N. crassa* (NCU03699, *znf-13*) and was re-named *res-1* (55). Here we show that a deletion of *res1* in *T. thermophilus* specifically affected conidiation in submerged cultures and therefore focused our efforts on the characterization of *res1*.

**Fig 3:**
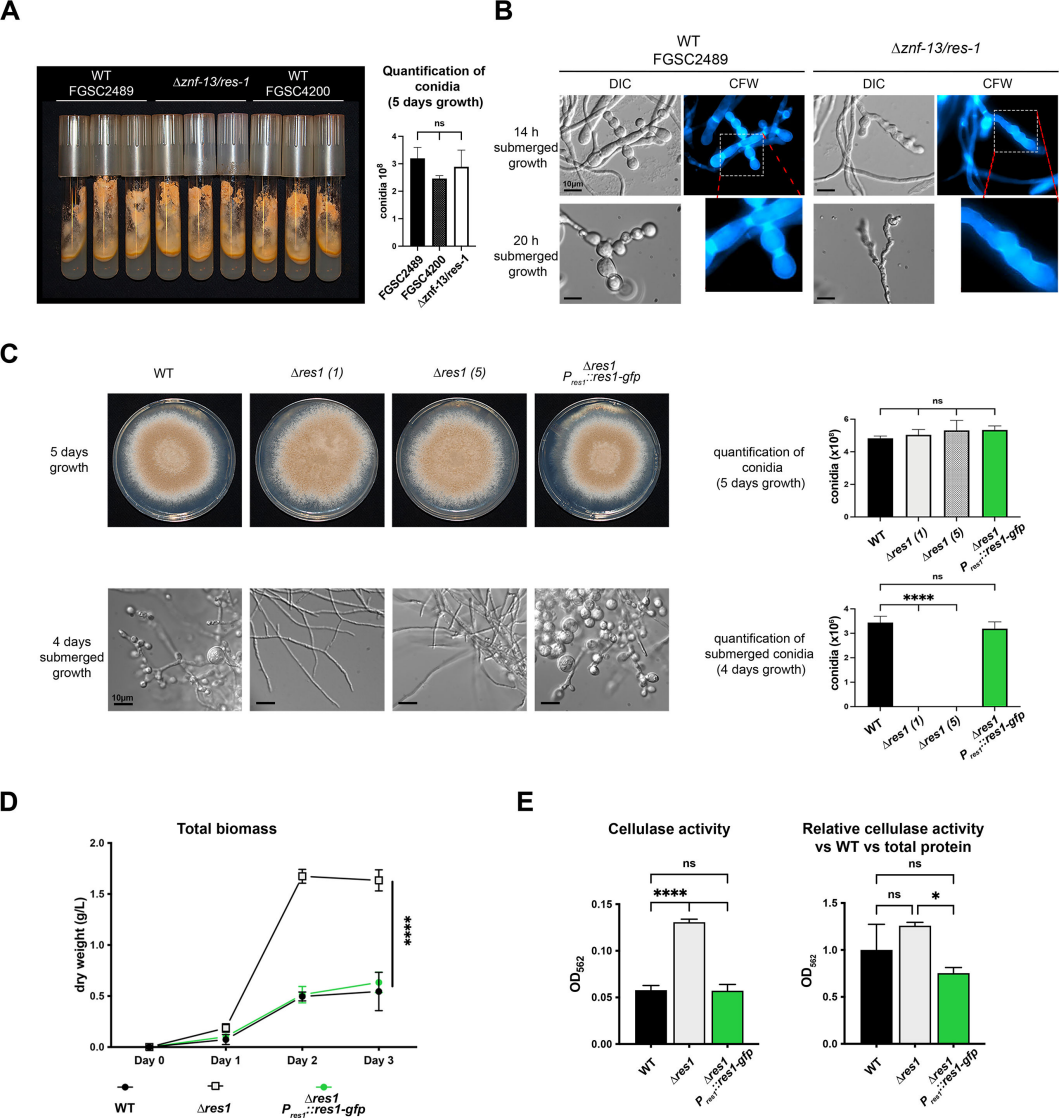
The conserved TF RES1 is required for conidia formation in submerged cultures of *N. crassa* and *T. thermophilus*. (A) VMM agar slant tubes of *N. crassa* WT (FGSC2489, FGSC4200) and ∆*res-1* mutant (FGSC11130) and the corresponding conidia quantification. ns = non-significant, using a one-way ANOVA. (B) Representative microscopy pictures of three biological replicates *N. crassa* WT (FGSC2489) and the ∆*res-1* mutant (FGSC11130) after 14 h and 20 h submerged growth in PCM medium. Cell walls were stained with calcofluor white (CFW) (1 µg/mL). (C) *T. thermophilus* WT and mutants *∆res1* (1), *∆res1* (5), and ∆*res1 P_res1_::res1-gfp* were grown on VMM (39) agar plates and submerged in PCM (30) with conidia quantification under both conditions. ns = non-significant, ****P ≤ 0.0001 using a one-way ANOVA with a Tukey’s post hoc test. (D): *T. thermophilus* WT, ∆*res1* (1), and ∆*res1 P_res1_::res1-gfp* strains were grown in a main-culture medium and the total biomass was determined on day 1, day 2, and day 3. ****P ≤ 0.0001 using two-way ANOVA with a Tukey’s post hoc test. (E) Cellulase activity of *T. thermophilus* WT (ATCC42464), *∆res1* (1), and *∆res1 P_res1_::res1-gfp* strains. Strains were directly inoculated and grown in VMM 2% Avicel for 48 h and cellulase activity was measured in the supernatant. Normalization for biomass in all three cultures was performed by quantifying the total extracted protein from the collected mycelium. ns = non-significant, ****P ≤ 0.0001 using a one-way ANOVA with a Tukey’s post hoc test.

The *T. thermophilus res1* locus encodes a Zn2Cys6 transcription factor with four zinc finger domains (Fig. S6A) with 73.9% amino acid (aa) identity to its *N. crassa* homolog *znf-13/res-1* (Fig. S6B). Deletion of either the *N. crassa res-1* gene or the *T. thermophilus res1* homolog caused a significant increase in xylanase, endoglucanase, and exoglucanase activity under cellulolytic conditions (5, 55). To assess whether a deletion of this TF affected conidiation, we first assessed morphological differences of the *N. crassa* ∆*res-1* mutant versus the WT strains (FGSC2489 and FGSC 4200) when grown on VMM plates (39); no morphological differences or differences in conidia production were observed (Fig. 3A). When cultures were grown submerged in PCM, conidiation was visible in the *N. crassa* WT strain at 20 h, but the ∆*res-1* mutant failed to form double-septa and only showed minor constrictions and lacked conidia formation at 20 h (Fig. 3B; Fig. S1). These data suggested that ∆*res-1* has a role in conidia formation in *N. crassa* in submerged cultures but was not essential for conidiation on solid media.

We generated two independent *T. thermophilus* ∆*res1* mutants (mutants 1 and 5) and a strain in which we complemented the *res1* deletion with an ectopic integration of *P_res1_::res1-gfp* (Fig. S3A; ∆*res1* C). Both *T. thermophilus* ∆*res1* deletion mutants and the *res1* complemented strain formed conidia on VMM agar plates and were indistinguishable from WT cultures in morphology and conidial production (Fig. 3C; top row). While the *T. thermophilus* WT and *res1* complemented strain formed conidia in submerged cultures, the ∆*res1* mutants showed no conidia formation over the time course (Fig. 3C; lower row). Because cells lacking *res1* continued to form mycelia while WT cells switched to conidia formation, we hypothesized that more fungal biomass would accumulate in the *T. thermophilus* ∆*res1* cultures. Indeed, after 3 days of growth, the ∆*res1* mutant showed significantly more fungal biomass compared to WT and the *res1* complemented strains (Fig. 3D).

It was reported that a *res1* deletion strain in *T. thermophilus* increases CAZyme secretion (5); we therefore measured the cellulase activity in the ∆*res1* mutant. A 2.5-fold increase in cellulase activity in the *T. thermophilus* ∆*res1* strain was observed as compared to the WT and *res1* C strains (Fig. 3E), similar to what was previously reported (5). However, when cellulase activity was normalized to fungal biomass, the increased cellulase activity could be explained by the increased mycelial growth in submerged cultures of the ∆*res1* strain. These data suggest that deletion of *res1* does not lead directly to a hypersecretion phenotype but instead increased hyphal biomass and therefore the secretion of proportionally more CAZymes.

### *T. thermophilus* Res1 is involved in a variety of metabolic-related processes

**Fig 4:**
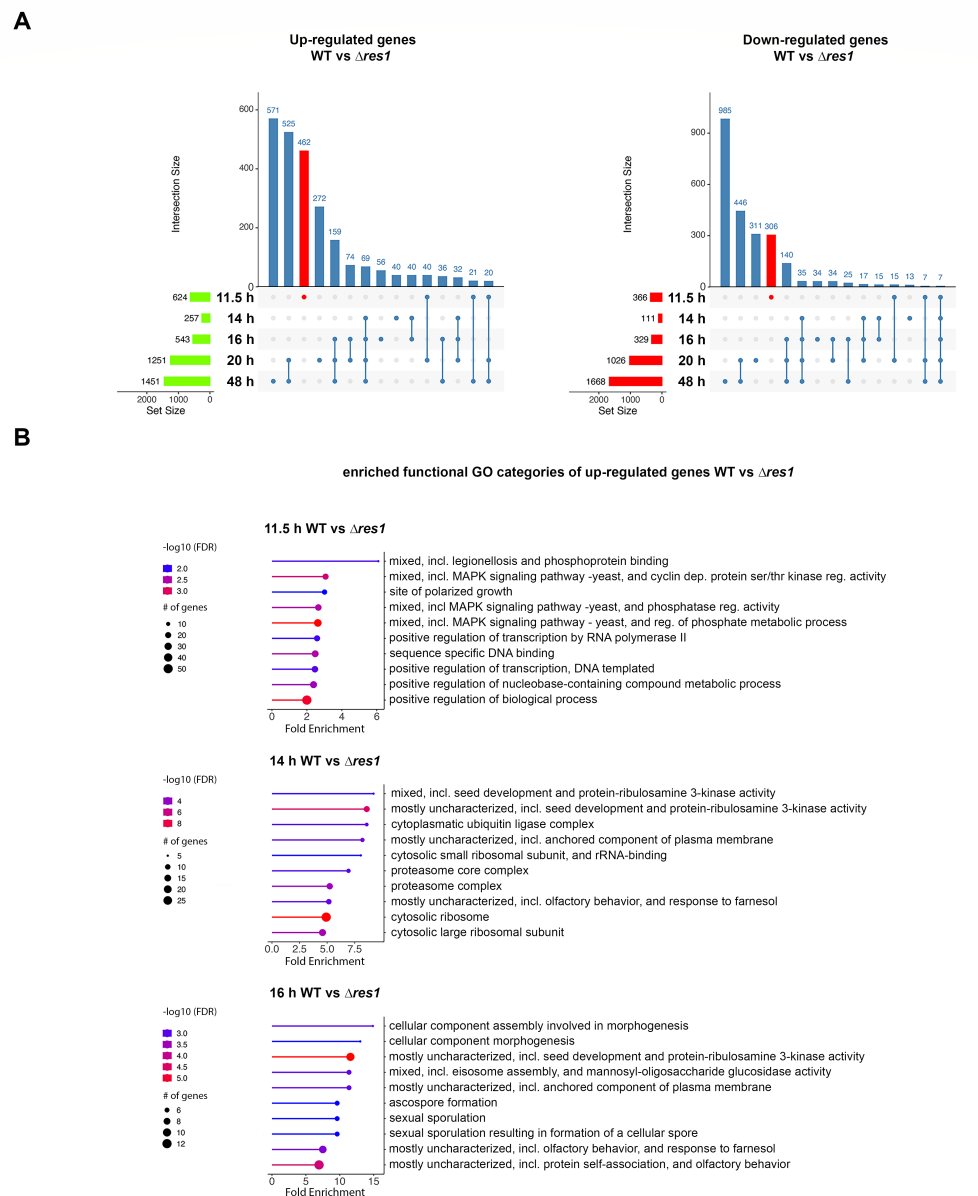
*T. thermophilus* Res1 regulates many genes during hyphal growth and conidial formation in submerged cultures. (A) The UpSet plot of intersecting upregulated (top) and downregulated (bottom) genes between *T. thermophilus* WT and the *∆res1* mutant at the indicated time points. Bars on the left indicate the total number of upregulated and downregulated genes at the time point and bars on top show the number of shared upregulated and downregulated genes at the indicated timepoints. (B) Enrichment analysis of GO terms at the timepoints 11.5 h, 14 h, and 16 h comparing WT vs the ∆*res1* mutant (Data set S6). Displayed are the fold enrichment, number of genes, and FDR of the 10 highest enriched categories using ShinyGO (v0.8; http://bioinformatics.sdstate.edu/go/). Displayed genes had an FDR ≤ 0.05.

To better understand the role of *res1* in *T. thermophilus* development in submerged cultures, we analyzed a time course of the transcriptome of WT and the ∆*res1* mutant at 11.5 h, 14 h, 16 h, 20 h, and 48 h (Data set S4). We identified 3,212 genes that were differentially expressed between WT and the ∆*res1* mutant by at least twofold (FDR < 0.05) (Data set S5). When comparing differentially expressed genes between the WT with the ∆*res1* mutant at 11.5 h, before phenotypic differences between WT and the ∆*res1* mutant were observed microscopically, 462 genes showed increased expression levels and 306 genes that showed downregulation uniquely at this timepoint in the WT as compared to the ∆*res1* mutant cultures (Fig. 4A).

At the 14-h time point, *res1* was upregulated by almost 3.5-fold as compared to 11.5 h (Data set S2, WT14 h_vs_11.5 h). At the 14-h time point, 257 genes showed upregulation and 111 genes showed downregulation. At later time points, an increase in the number of differentially regulated genes (1,451 upregulated and 1,668 downregulated genes) was observed (Data sets S4 and S5). To further understand the biological processes that were differentially regulated between WT and the ∆*res1* mutant across the time course in submerged cultures, we performed functional category enrichment.

At 11.5 h, the highest fold enrichment in GO categories in the WT strain compared to the ∆*res1* mutant was in MAPK signaling pathways and cyclin-dependent protein serine/threonine kinase regulator activity (3.1-fold enrichment, 0.0015 FDR), polarized growth (threefold enrichment, 0.011 FDR), and (positive) regulation of transcription by RNA polymerase II (2.6-fold enrichment, 0.009) (Fig. 4B; Data set S6). The highest enrichment in downregulated genes was mRNA splicing related (13.4-fold enrichment, 2.04e−06 FDR), mitochondrial respiratory chain complex assembly, (7.3-fold enrichment, 8.29e−06 FDR), and pre-ribosome assembly (6.9-fold enrichment, 1.38e−05 FDR). At 14 h, the highest fold enrichment in GO categories in the WT strain as compared to the ∆*res1* mutant were seed development and protein-ribulosamine 3-kinase activity (9.2-fold enrichment, 0.0025 FDR), cytosolic small ribosomal subunit and rRNA-binding (8.1-fold enrichment, 0.005 FDR), and proteasome core complex (6.9-fold enrichment, 0.0012 FDR) (Fig. 4B; Data set S6). In the downregulated genes, the highest enrichment was in categories involved in chromosome and microtubule organization (2-fold enrichment, 0.027 FDR) and amino acid transport (2.4-fold enrichment, 0.027 FDR) (Data set S6).

At 16 h, the WT showed the highest fold enrichment in GO categories involved in morphogenesis (14.9-fold enrichment, 0.00088 FDR), seed development and protein-ribulosamine 3-kinase activity (11.6-fold enrichment, 3.92e−06 FDR), sexual spore formation (9.6-fold enrichment, 0.0016 FDR), and olfactory response to farnesol (7.5-fold enrichment, 0.00059 FDR) (Fig. 4B; Data set S6). The highest enrichment in the downregulated genes was in the GO categories related to vacuolar transport (fourfold enrichment, 0.0072 FDR), cell wall related, including chitin binding, and fungal-type cell wall organization or biogenesis, (3.75-fold enrichment, 0.01 FDR) and carbohydrate metabolism (9.4 fold-enrichment, 0.01 FDR) (Data set S5). Overall, GO category analyses identified pathways predicted to be involved in phenotypical differences between the WT strain and the ∆*res1* mutant, which continued to grow vegetative hyphae in submerged cultures while the WT strain underwent the developmental process of conidiation.

### Over-expression of *res1* in *T. thermophilus* results in pre-mature conidiation in plates and submerged cultures

**Fig 5:**
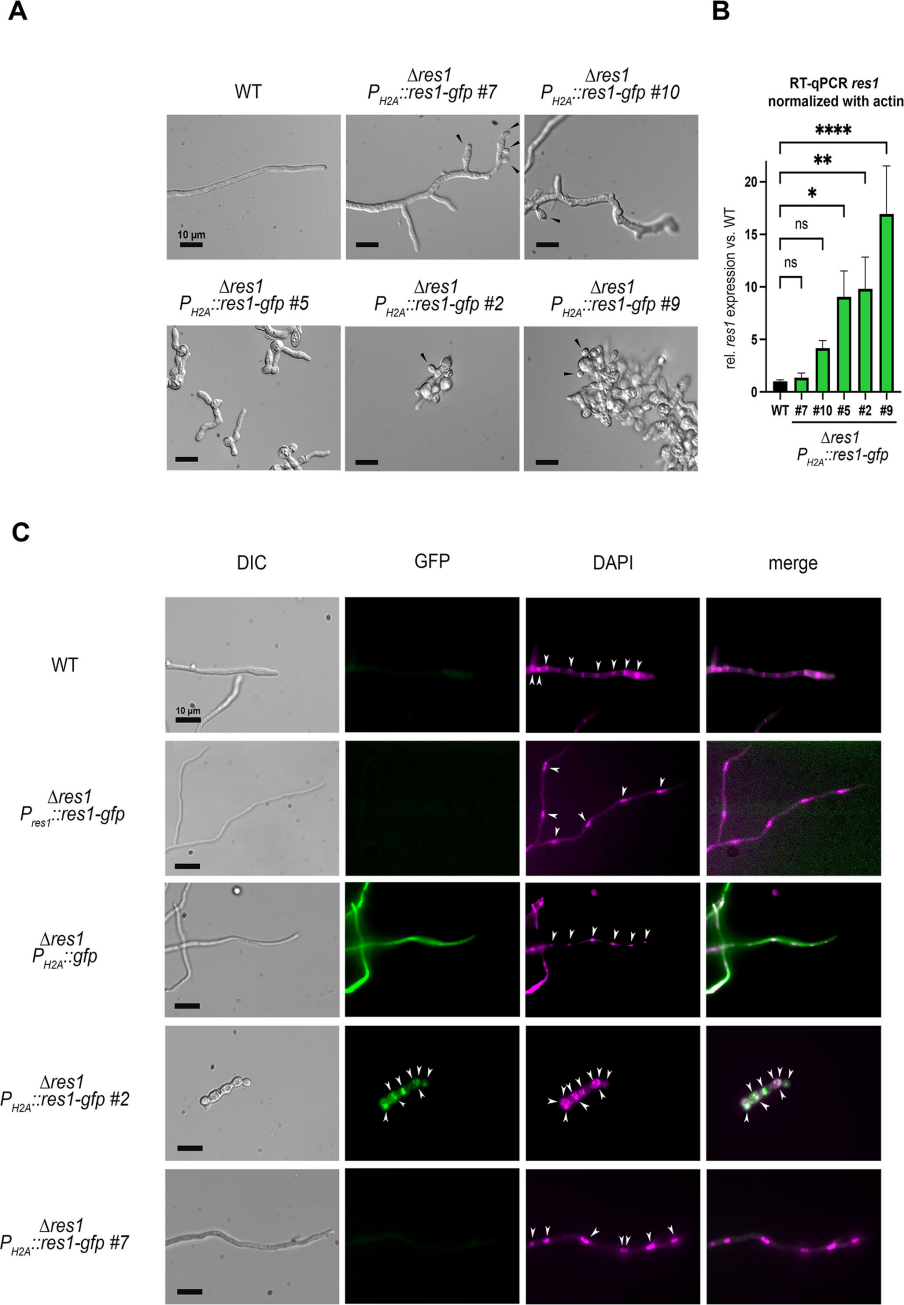
Overexpression of *res1* causes premature conidiation in *T. thermophilus*. (A) Representative microscopic images of *T. thermophilus P_H2A_::res1-gfp* and WT strains after 12 h of submerged growth in PCM (30). No conidiation is visible in the WT strain while *res1* overexpression strains show conidia (black arrows). Scale bar: 10 µm. For plate phenotypes, see Fig. S7A. (B) RT-qPCR of *res1* mRNA expression in *P_H2A_::res1-gfp* strains relative to *res1* expression in the WT strain after 12 h of growth in submerged cultures. ns = non-significant, *P ≤ 0.05, **≤ 0.001,****P ≤ 0.0001 using a one-way ANOVA with a Tukey’s post hoc test. (C) Fluorescence microscopic images of WT, control strains, ∆*res1 Pres1::res1-gfp*, and *∆res1 P_H2A_::gfp,* and ∆*res1 P_H2A_::res1-gfp* #2 and #7 grown in PCM. Nuclei indicated by white arrows, scale bar 10 µm.

To assess whether Res1 directly regulates conidiation, in submerged cultures, we evaluated the phenotype of strains overexpressing *res1*. Plasmids were constructed with *res1* fused to *gfp* (*res1-gfp*) or with a V5-epitope tag (*res1-V5*) under the control of the strongly expressed, constitutive histone 2A (H2A) (30) promoter or the native *res1* promoter (Materials and Methods). As a control, a third plasmid was constructed expressing cytoplasmic *gfp* under the regulation of the H2A promoter. The constructs were transformed in the *T. thermophilus* ∆*res1* background and integrated ectopically. We obtained several transformants for both *res1* OE constructs (Fig. 5A; Fig. S7). As evaluated by RT-qPCR, strain *P_H2A_::res1-gfp* #7 had the lowest *res1* mRNA expression level (Fig. 5B; top) relative to WT. In submerged cultures in PCM (30), the *P_H2A_::res1-gfp* #7 strain began conidiating at 12 h, when the WT strain was still filamentous (Fig. 5A). Strains with higher *res1* expression, such as *P_H2A_::res1-gfp* #10, also showed conidiation in submerged cultures earlier than the strain WT but also displayed growth defects (Fig. 5A). Importantly, strains with the highest *res1* expression levels had stunted hyphal growth and showed conidial production on very short hyphae with occasional budding on swollen, germinated conidia (*P_H2A_::res1-gfp* #9) (Fig. 5A). The *res1-V5* overexpression strains also showed stunted growth and premature conidiation in submerged cultures (Fig. 5A; Fig. S7A). On plates, the *res1-V5* tagged over-expression strains also showed premature conidiation starting as early as 24 h after plating spores (Fig. S7A). After 48 h, the colony edges of all overexpression strains showed conidia production, in contrast to the WT, which only started to form conidia at the center of the colony. In addition, defects in polarized growth were visible in the mutants while the WT formed straight hyphae with little bending (Fig. S7A). These data indicate that over-expression of *res1* leads to stunted hyphal growth and premature conidiation on plates and in submerged cultures.

The *T. thermophilus res1* locus is predicted to encode a TF, but localization of the Res1 protein has not been reported in either *N. crassa* or *T. thermophilus*. In *T. thermophilus,* we did not detect a GFP fluorescence signal when GFP-tagged Res1 was expressed under its native promoter (Fig. 5C), but a GFP signal was observed in strains expressing cytoplasmic GFP under the regulation of the H2A promoter (30). In strains over-expressing *res1* (*P_H2A_::res1-gfp* #2), we observed co-localization of Res1-GFP- and DAPI-stained nuclei (Fig. 5C), consistent with its function as a TF. In strains such as *P_H2A_::res1-gfp* #7, we observed both normal hyphae and stunted hyphae that showed premature conidiation, suggesting that some of the hyphae “escape” from the Res1 over-expression phenotype. Indeed, when the localization of Res1-GFP was assessed in this strain, no Res1-GFP signal was observed in the morphologically normal hyphae (Fig. 5C). However, hyphae in the *P_H2A_::res1-gfp* #7 strain that showed stunted growth and premature conidiation showed nuclear co-localization of Res1-GFP and DAPI (Fig. S7C).

### RNA-Seq analysis of the *res1* over-expression strains

**Fig 6:**
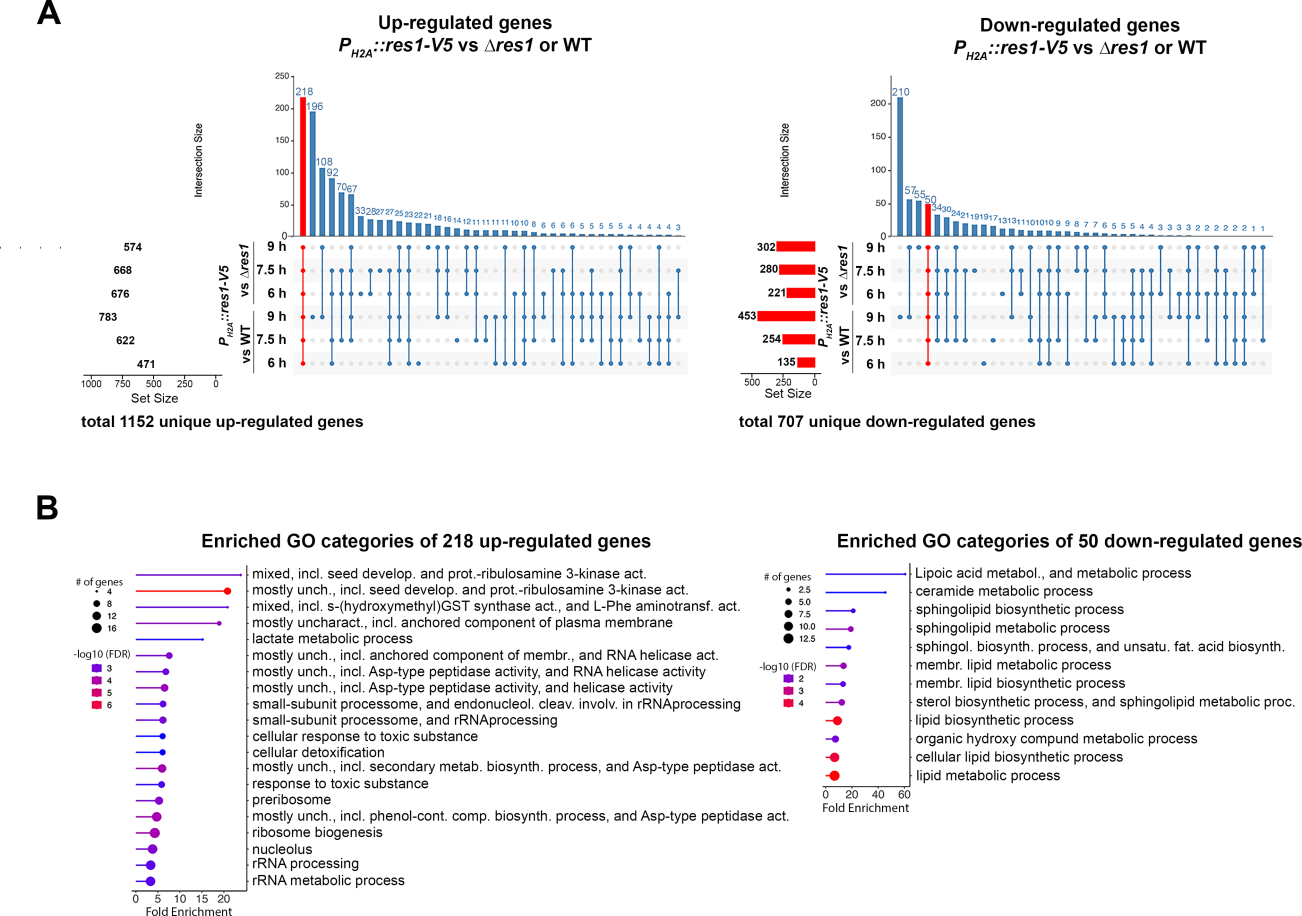
In *T. thermophilus* Res1 directly regulates genes with predicted roles in conidiation and nutrient sensing. (A) UpSet plots of upregulated and downregulated genes between the ∆*res1 P_H2A_::res1-V5* strain and either the ∆*res1* mutant or WT strain at 6 h, 7.5 h, and 9 h in submerged cultures grown in PCM. (B) Functional category enrichment of shared genes between ∆*res1 P_H2A_::res1-V5*, the ∆*res1*, or WT strains at all time points. Displayed are the fold enrichment, number of genes, and FDR in the highest enriched categories with FDR ≤ 0.05 using ShinyGO (v0.8) (33).

To find possible targets of Res1 in *T. thermophilus,* we used the ∆*res1 P_H2A_::res1-V5* #13 strain, which had the highest level of *res1* expression (Fig. S7B) to conduct transcriptional profiling and chromatin immunoprecipitation sequencing (ChIP-Seq) experiments. We first compared transcriptional profiles of WT, ∆*res1,* and ∆*res1 P_H2A_::res1-V5* strains at timepoints just after germination and in early hyphal development after 6, 7.5, and 9 h growth in PCM (Fig. S8A; Data set S4). The WT strain and the ∆*res1* mutant showed fewer differentially regulated genes at early time points (at 6 h: 99 genes; 7.5 h: 15 genes; 9 h: 145 genes, Fig. S8B) as compared to the *res1* overexpression strain, which had 1,152 unique upregulated and 707 unique downregulated genes (Fig. 6A; Data set S4). The intersection of all timepoints of the *res1* overexpression strain showed 218 upregulated and 50 downregulated genes as compared to the WT strain and the ∆*res1* mutant that was shared over all timepoints (Fig. 6A, red bars).

We used GO category enrichment (33) to further understand the functions and pathways of these gene sets. Of the 1,152 genes that were uniquely upregulated in the *res1* overexpression strain at any timepoint, the highest fold enrichment was found with ribosome and RNA-related categories with most genes in the categories of small subunit processome, ribosome biogenesis, and rRNA metabolic process (Fig. S9). Of the 218 genes that were upregulated in the *res1* overexpression strain across all three timepoints, the highest enrichment was in seed development-related categories, metabolism of glutathione S (GST) and aspartate (Asp), and ribosomal-related categories (Fig. 6B).

Of the 707 that were uniquely downregulated in the *res1* overexpression strain at any timepoint, the highest fold enrichment categories were ribosome-related categories, siderophore metabolic processes, G-protein and division septum related, and organic acid transmembrane transporter-related categories (Fig. S9). Of the 52 genes that were downregulated in the *res1* overexpression over all three timepoints, the highest enrichment was found in lipoic acid metabolism, ceramide metabolic process, sphingolipid synthesis, and metabolism and other (membrane) lipid related processes (Fig. 6B).

### ChIP-Seq to identify direct Res1 targets and motif discovery

To further define direct targets of Res1 in *T. thermophilus*, ChIP-Seq was conducted early in development at the 16-h time point in ∆*res1 P_H2A_::res1-V5* #13, ∆*res1 P_res1_::res1-V5*, and WT submerged cultures in VMM confirming the start of conidiation in this medium via microscopy. Anti-V5 antibodies were then used to immunoprecipitate epitope-tagged Res1 bound to chromatin. We identified 30 peaks in the Res1 ChIP pulldown that were not present in the untagged WT negative control with a median distance of 1.3 kbp to the translational start site. Of these, 25 peaks were upstream of only one gene with 5 peaks upstream of two genes transcribed in opposite directions. Four outlier peaks were 6.4, 8.3, 8.7, and 11 kbp upstream of the translational start site, resulting in a total of 35 putative target genes (Data set S7).

**Fig 7:**
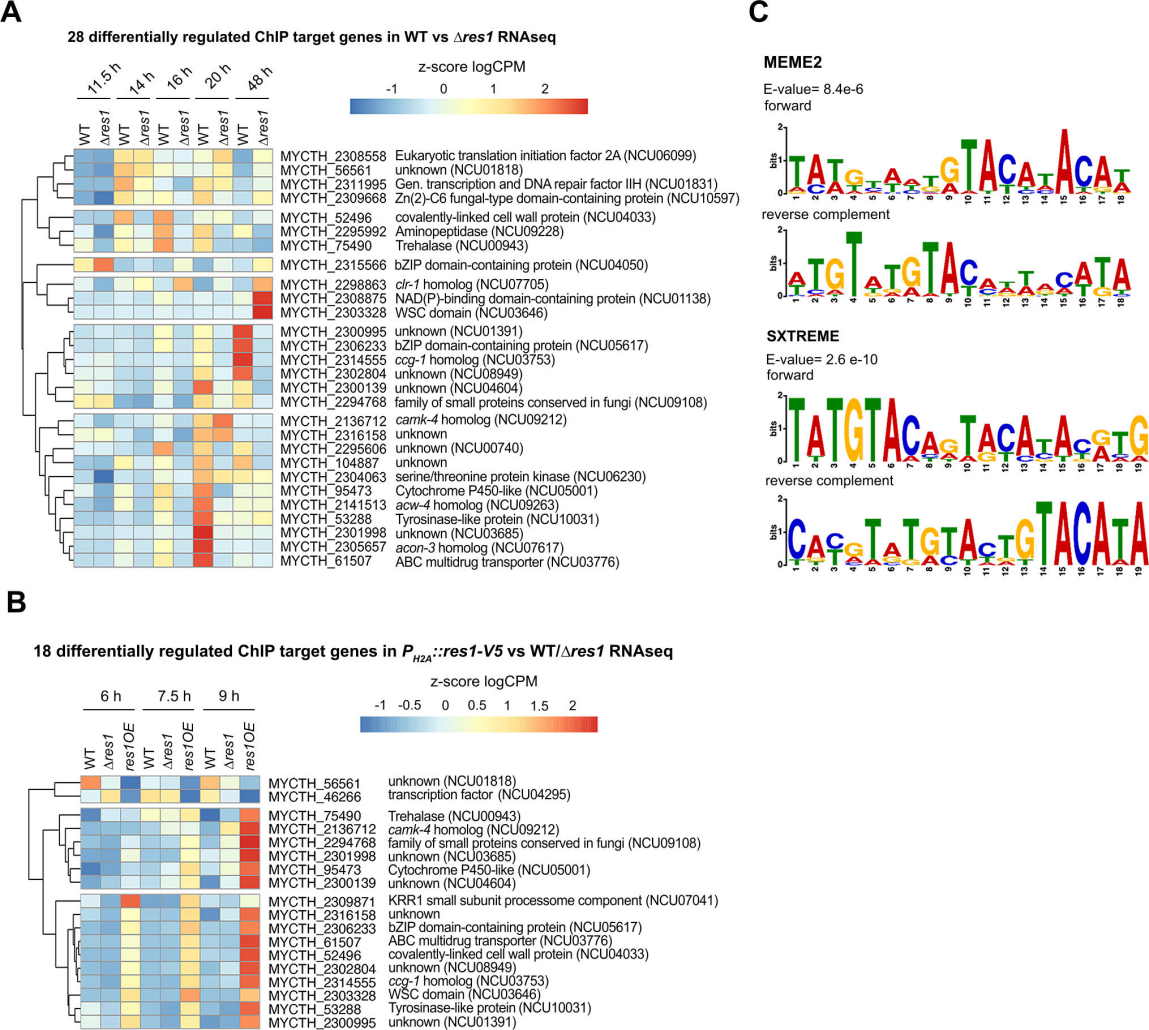
Res1 directly regulates genes with predicted roles in conidiation and nutrient sensing. (A) Heatmap of 28 genes that have a ChIPseq peak upstream of the translational start site and that were differentially regulated in the RNAseq data set comparing WT vs ∆*res1*. Heatmap shows z-score from log10 counts per million (logCPM). (B) Heatmap of 18 genes that have a ChIPseq peak upstream of the translational start site and that were differentially regulated in the RNAseq data set comparing *P_H2A_::res1-V5* vs ∆*res1* and WT. Heatmap shows z-score from log10 counts per million (logCPM). (C) Res1-binding motif obtained from MEME suite v5.5.5 using ChIPseq binding peak sequences 4 kbp or less from the TSS. Shown are the motifs predicted by MEME (E-value 8.4 × 10^−6^, 18 bp) and by STREME (E-value 2.6 × 10^−10^, 19 bp) with a SEA rank of 2 and 4, respectively.

Of the 35 Res1 target genes, 28 were significantly differentially regulated between WT and *∆res1* strains over the time course of growth in submerged cultures (Fig. 7A; Data set S2, S6); 26 of these 28 genes have a homolog in *N. crassa*. One gene, a homolog of NCU04050 (MYCTH_2315566), which encodes the Cross Pathway Control (*cpc-1*) transcriptional regulator in *N. crassa,* showed elevated expression levels in the ∆*res1* mutant relative to the WT strain, particularly at the 11.5-h time point (Fig. 7A; Data set S7). A homolog to a *N. crassa* TF gene encoding a cellulose regulator 1 (*clr1;* MYCTH_2298863) was also upregulated in the ∆*res1* mutant relative to WT, except for at the 11.5-h time point. Two additional genes, MYCTH_2308875, encoding a predicted NAD(P)-binding domain protein and a gene encoding a protein with a WSC domain (MYCTH_2303328) also showed significantly increased expression levels at the 48-h time point in the ∆*res1* mutant relative to the WT strain (Fig. 6C; Data set S7).

Of the remaining 24 differentially regulated Res1 target genes, 20 genes showed a significant increase in expression levels in the WT strain during the time course in submerged cultures relative to the ∆*res1* mutant (Fig. 7A; Data set S4, S7). Of these 20 genes, three Res1 target genes showed the highest expression in WT at 16 h relative to the ∆*res1* mutant, including one gene predicted to encode trehalase (MYCTH_75490); a trehalase mutant in *A. niger* was affected in conidiation (56), a gene encoding a predicted aminopeptidase; aminopeptidases has been reported to be involved in promoting conidial viability (57) and MYCTH_52496, which encodes a homolog to *N. crassa wal-4* (NCU04033), a predicted covalently-linked cell wall protein (https://fungidb.org/fungidb/app). MYCTH_52496 was one of the 25 most highly expressed genes during early conidiation (Data set S2, 14 h_vs_11.5 h). A second set of 11 genes showed significantly increased expression levels at the 14, 16, and 20 h time points (Fig. 7A), with nine genes showing the highest expression level at the 20-h time point. Three of these nine Res1 target genes have potential functions in conidiation, including the homolog of the *N. crassa* conidiation regulator *acon-3* (MYCTH_2305657) (19, 44), a homolog to a gene encoding a tyrosinase-like protein (MYCTH_53288), which is involved in conidiation in *Magnaporthe oryzae* (58) and a gene encoding a homolog to a cell wall protein (*acw-4*; MYCTH_2141513) identified in *N. crassa* conidial cell walls (59, 60) (Fig. 7A). The remaining six genes included three genes encoding proteins of unknown function (MYCTH_2295606; MYCTH_104887; MYCTH_2301988), a gene predicted to encode a serine threonine protein kinase (MYCTH_2304063), a cytochrome P450-like protein (MYCTH_ 95473), which is a homolog of NCU05001, an ent-kaurene oxidase, which is part of a secondary metabolite cluster that is upregulated during conidiation in *N. crassa* (61) and an ABC multidrug transporter gene (MYCTH_61507), which is a homolog of NCU03776, which is involved in azole resistance (62) (Data set S7); MYCTH_61507 was a member of the 25 mostly highly expressed genes at the onset of conidiation (Data set S2). A third set of six Res1 target genes showed the highest level of expression in the WT time course at 48 h, including three genes encoding proteins of unknown function (MYCTH_2300995; MYCTH_2302804; and MYCTH_2300139), a gene encoding a member of conserved small proteins in fungi (MYCTH_2294768), a predicted bZIP TF (MYCTH_2306233), and a homolog of the clock-regulated gene, *ccg-1* in *N. crassa* (MYCTH_2314555).

In all, 18 of the 35 Res1 target genes also showed an increase in expression levels in the *res1* OE expression strain at least one time point. In all, 16 of the 18 genes showed an increased level of expression across all three time points with 14 genes showing their highest expression level at the 9-h time point (Fig. 7B; Data set S4 and S7). Only one gene (MYCTH_2309871) encoding a *KRR1* homolog showed the highest expression level at the earliest time point (6 h) (Fig. 7B). In *Saccharomyces cerevisiae*, *KRR1* encodes an essential gene that is part of the 90S preribosome and is involved the ribosomal small subunit biogenesis (63). Two Res1 target genes, MYCTH_56561, encoding a protein of unknown function, and MYCTH_46266, encoding a predicted TF (NCU04295; *tcf-7* in *N. crassa*), showed a decrease in expression level in the *res1* OE strain relative to the WT strain across all three time points (Fig. 7B).

To identify a Res1-binding motif, we used the 27 ChIP-seq peaks that were between 30 bp and 3.7 kbp from a predicted translational start site using the motif-based sequence analyses tools, MEME2 and SXTREME suites (https://meme-suite.org/meme/). The two best motif matches were TAWGYWHKGTACATACA and TATGTACAGTACATACRTG, which both had a TATG (position 1 to 4)/ATAC (position 15–12) palindrome sequence (Fig. 7C). The TAWGYWHKGTACATACA motif was present in the promoters of 20 of the 35 possible target genes (17 peaks). These genes included two genes (MYCTH_2129400 and MYCTH_90516) that were not differentially expressed in any of the tested conditions, suggesting they may be regulated by Res1 at a time point or under a condition that we did not test. The TATGTACAGTACATACRTG motif was present in the promoters of 10 of the 35 potential target genes (eight peaks) (Data set S7).

## DISCUSSION

Here, we investigated the morphological and transcriptional regulation of conidiation in submerged cultures of the industrially relevant species, *T. thermophilus*. From this approach, we identified the TF *res1*, which when mutated, completely abolished conidiation of *T. thermophilus* in submerged cultures, but not when exposed to air on plates, a phenotype that is similar to the *res-1* mutant in *N. crassa* (Fig. 3B). To our knowledge, *res-1/res1* is the first regulator of conidiation that separates developmental aspects associated with conidiation on plates versus submerged cultures. These data indicate that cultures exposed to air can bypass the need for RES-1/Res1, demonstrating differential regulation of conidiation under these two environmental conditions. Our data support the hypothesis that the switch to conidiation in submerged cultures leads to a reduction of fungal biomass, resulting in a reduction of enzyme/protein production during fermentation, as well as producing unwanted asexual propagules under these conditions. Our data showed that the increased cellulolytic enzyme activity of the *T. thermophilus* ∆*res1* mutant that was previously reported (5, 55) is likely due to the increased biomass accumulation that occurs when cells do not make the switch from hyphal growth to conidiation under submerged culture conditions.

Our expression profiling data comparisons between the *T. thermophilus* WT, the *∆res1* mutant, and the *res1* over-expression strain revealed processes that are regulated either directly or indirectly by Res1. Indeed, even before conidiation was apparent, there were more than 500 upregulated and 300 downregulated genes between WT versus the ∆*res1* mutant (Fig. 4A). We identified homologs to known regulators of conidiation in *N. crassa* that increased in expression as conidiation progressed in submerged cultures. These included a homolog to *ada-6*, whose deletion affects conidiation (37), *gnb-1* (38), in which mutations result in inappropriate conidiation in submerged cultures, *aconidial-2 (acon-2*) and *acon-3* (18), which regulate conidial constrictions during conidiation (11), *asm-1,* which regulates conidial germination and sporulation pattern (64)*,* and two TFs that regulate conidial separation *csp-1* and *csp-2* (20). In *N. crassa*, ADA-6 positively regulates the conidiation-specific genes *eas*, *con-6*, *con-8*, *con-10*, *con-13*, *pcp-1*, and NCU09357 (37). In *T. thermophilus,* homologs to *con-6, con-8,* and *con-10* showed very high expression levels late during the conidiation process (48 h), which mirrors the expression pattern observed for the *con* genes during *N. crassa* conidiation (40, 41). These data suggest that, even though morphological aspects associated with conidiation differ between *N. crassa* and *T. thermophilus* (Fig. S1), regulatory aspects show conservation. In *N. crassa*, analysis of *acon-2* and *acon-3* mutants in *fl* over-expression strains provided evidence that FL acts upstream of ACON-3 and downstream of ACON-2 (21). These data in *N. crassa* are consistent with our expression profile data of the *T. thermophilus acon-2* and *acon-3* homologs in submerged cultures (Fig. 2A). Importantly, the *T. thermophilus acon-3* homolog was identified as a direct target of Res1 via ChIP-Seq (Data set S7).

In *N. crassa*, strains carrying a disruption of the TF *fluffy* fail to form minor and major constrictions in aerial hyphae and are aconidial on plates and in submerged cultures (44, 53). However, unlike *N. crassa*, the *T. thermophilus* ∆*fl* mutant produced conidia on plates and in submerged cultures grown in VMM. However, in PCM, submerged cultures of the *T. thermophilus* ∆*fl* mutant failed to form conidia, like the *N. crassa* ∆*fl* mutant. It is not obvious what differences in VMM versus PCM mediated this difference in sporulation in submerged cultures. In *N. crassa*, expression of *fl* is regulated by a number of environmental factors and is induced under nitrogen starvation (21). The *T. thermophilus fl* homolog showed early upregulation (14 h) in two of the three biological replicates, suggesting that FL may also play a role in integrating nutritional/environmental signaling in this species to regulate conidiation when cells are submerged.

The Res1 over-expression strain showed premature conidiation in submerged cultures and on plates (Fig. 5A; Fig. S7A). However, morphological changes seen in the *res1* overexpression strain relative to WT suggest that integration of Res1 pathways with other developmental programs associated with vegetative and polarized growth is needed for WT growth morphology and asexual development. In *N. crassa*, conidiation is induced by environmental conditions, such as transfer from liquid to an air interface, nutrient limitation, CO_2_ levels, light, and the circadian clock (17). The regulation of conidiation by light requires the photoreceptors WC-1 and WC-2, which are two PAS domain-containing transcription factors, which are also required for light entrainment of the circadian clock (65, 66). A homolog of *wc-1* in *T. thermophilus* showed upregulation at 14 h during growth in submerged cultures (Table S2), while a homolog of *N. crassa adv-1,* which is a direct target of the WC1/WC2 complex (22), was upregulated at the 14-h time point (Table S2). In addition to *wc-1* and *adv-1*, a number of other TFs that affect conidiation in *N. crassa* were upregulated at 14 h in submerged cultures of *T. thermophilus* (Table S2), including mutants phenotypically characterized in screens of TF deletion strains (67). These predicted TFs included *ada-10, vad-5, tah-5, ada-1,* NCU00019, NCU03070, *tah-1*, *sah-4*, NCU03417, and NCU06145 (68) (Table S2). Subsequent work on *vad-5* in *N. crassa* showed that a ∆*vad-5* mutant showed downregulation of important regulators of conidiation, such as *fl, ada-6, rca-1,* and *eas* (69).

The clock-controlled gene *ccg-1,* with unknown biochemical function, has been used extensively to assess the regulation of both light and asexual development in *N. crassa* (70–73). In this study, *ccg-1* was identified as a direct target of *T. thermophilus* Res1 and was also highly expressed in the *res1* over-expression strain. Other genes, whose products have been implicated in conidiation or conidial viability in other species (trehalase and tyrosinase), were also bound by Res1 and were highly expressed in the *res1* over-expression strain. These data indicate that Res1 plays a direct role in the regulation of a set of genes important for asexual development. These data provide a rich resource to evaluate conserved and divergent aspects associated with regulatory and transcriptional regulation associated with conidiation under different environmental regimes.

This study has revealed genes in *T. thermophilus* and *N. crassa, res-1/res1* that specifically affect conidiation in submerged cultures but that do not affect conidiation on plates. For *T. thermophilus*, this has potential biotechnological implications for increasing biomass under fermentation conditions in addition to preventing the production of asexual spores in submerged cultures. The commonalities in the regulatory landscape of environmental/nutrient sensing and conidiation development in *T. thermophilus* and *N. crassa* provide an avenue to further explore evolutionary features associated with conidiation in filamentous ascomycete species.

## MATERIALS AND METHODS

### Re-annotation of the *T. thermophilus* genome

To enrich the annotation of the *T. thermophilus* genome, we identified orthologous genes shared between *N. crassa* and *T. thermophilus* by OrthoFinder (31) and expanded the number of GO terms using eggNOG-mapper v2 (32) (Fig. S2).

### Microbial strains and culture conditions

*T. thermophilus* strain 42464 was obtained from the American Type Culture Collection. The ∆*ku80* strain MJK20.2 was generously provided by Vera Meyer (4). *N. crassa* strains FGSC 2489 (wild type), FGSC 4200 (wild type), FGSC 11130 (∆*znf-13*), and FGSC 11044 (∆*fl*) were obtained from the Fungal Genetics Stock Center (FGSC) (74). *T. thermophilus* strains constructed in this study have been deposited at the FGSC: ∆MYCTH_2296328 (FGSC 27325); ∆*ku80* ∆MYCTH_2071661 (FGSC 27324); ∆MYCTH_2302052 (FGSC 27323); ∆MYCTH_2302052 *P_res1_::res1-V5* (FGSC 27322); ∆MYCTH_2302052 *P_res1_::res1-gfp* (FGSC 27321); and ∆MYCTH_78277 (FGSC 27320).

Strains were grown at 37°C (*T. thermophilus*) or 30°C (for *N. crassa*) on VMM (39). For submerged culture experiments, VMM, complete medium (CM) (75), pre-culture medium (PCM), and main-culture medium (MC) were used (30). One-liter Erlenmeyer flasks with 300 mL of medium were used under light conditions for all RNA-Seq experiments. To obtain protoplasts, *T. thermophilus* was grown in CM. For plasmid propagation, *Escherichia coli* NEB 5-alpha and 100 µg/mL ampicillin or 50 µg/mL kanamycin for selection were used.

### Molecular techniques

Upstream and downstream regions of the genes of interest were amplified by PCR (Data set S8) from genomic DNA and fused by double-joint PCR (76) with a hygromycin cassette for MYCTH_2296328 and MYCTH_2302052 (*res1*). A split marker approach (77) was used for deletion of MYCTH_2071661 (*fl*) by Cas9 RNPs (4), an *amdS* split marker with the 5′ UTR and 5′ *amdS* and the 3′ UTR and 3′ *amdS* fragments were fused by double-joint PCR and used in equimolar ratios for transformation. Transformants were picked from selection media and verified by PCR (Fig. S3; Data set S8). To generate the complementation plasmid with *res1* expressed under the native promoter, pLYMT-1, the plasmid backbone (Kan^R^), a nourseothricin resistance cassette, the native *res1* promoter, the *res1* ORF, and *sfGFP* were amplified by PCR (Data set S8) and fused together using the HiFi DNA Assembly Cloning Kit (NEB). The *res1* overexpression plasmid pLYMT-4 was constructed by amplifying a 1,005 bp fragment of the H2A promoter, an industrially used promoter for protein overexpression (patent WO2017093451A1) (30), a 1 kb segment directly upstream of the TSS of MYCTH_112137, and fusing it with the pLYMT-1 backbone, *res1,* and *sfGFP* using the HiFi DNA Assembly Cloning Kit (NEB). Plasmid pFDMT-1 for V5 tagged *res1* under the H2A promoter control was constructed from the backbone as well as a second construct with the NAT1, H2A, and *res1-*V5 were amplified from plasmid pLYMT-4 using oligonucleotides (Data set S8) and fused using the HiFi DNA Assembly Cloning Kit (NEB). Plasmid pFDMT-2 for V5 tagged *res1* under the native *res1* promoter was constructed amplifying pFDMT-1 without the H2A promoter and amplifying the native promoter from gDNA using oligonucleotides (Data set S8) and fused using the HiFi DNA Assembly Cloning Kit (NEB).

Protoplasts of *T. thermophilus* were obtained as described by reference (75) and transformed with either purified PCR product (to create *fluG*, MYCTH_2296328*,* and *res1* deletion strains), *Bam*HI linearized plasmid (pLYMT-1 and pLYMT-4), or RNPs (*fluffy* deletion) as described by reference 4.

### RNA extraction and RNA-Seq

RNA was extracted as described (78). After precipitation, RNA was resuspended in RNase-free water and DNaseI (NEB) was digested and purified on Monarch Total RNA Miniprep Kit (NEB) columns as per the vendor’s instructions. RNA concentration and quality were assessed by Nanodrop (Thermo Fisher Scientific), agarose gel, and 2100 Bioanalyzer (Agilent) system. Library preparation and sequencing were performed by the Functional Genomics Laboratory (FGL, QB3 UC Berkeley), using Kapa Biosystems library prep reagents and NovaSeq (Illumina) systems.

Reads were trimmed with trimmomatic (V 0.39) and quality control of fragments was done with FastQC (V 0.11.09). Fragments were mapped on the chromosomes with HiSat2 (V 2.1.0) and turned into SAM files with samtools (V 1.8). Analysis of mapped reads and visualization was performed with a custom R (V 4.2.3) script using the libraries “edgeR,” “limma,” “statmod,” “UpSetR,” “pheatmap,” and “RcolorBrewer,” similar to Law et al. (79). Gene ontology (GO) enrichment was done using ShinyGo (V0.80) (33) using the *T. thermophilus* genome as background, FDR ≤ 0.05, and a minimum of 5 and maximum of 500 genes per GO category/pathway. Gene count filters were used for each GO term.

### Chromatin immunoprecipitation

Chromatin immunoprecipitation was done similar to that described in Ferraro and Lewis (80) with some modifications. WT, ∆*res1 P_res1_::res1-V5*, and ∆*res1 P_H2A_::res1-V5* were inoculated (10^6^ conidia/mL) and grown in VMM for 16 h until conidiation started in WT and *P_res1_::res1-V5* (confirmed microscopically). Cultures were then harvested with miracloth, washed twice with PBS, and incubated in 1% formaldehyde in PBS for 15 minutes. The reaction was quenched and processed as described (81), and finally, samples were flash-frozen in liquid nitrogen for later use. Samples were homogenized via douncing in ChIP lysis buffer (81) before sonication with a Covaris S220 sonicator. Samples were then centrifuged at 20,000 × *g*, 4°C, and an aliquot of the supernatant was used as “input.” The left-over supernatant “pull down” fraction was then pre-cleared with Protein G DynaBeads (Life Technologies) for 2 hours. V5-tagged proteins were recovered by incubating the supernatant with an α-V5 antibody (rabbit polyclonal [14440-1-AP], Thermo Fisher Scientific) and Protein G DynaBeads (Life Technologies) overnight on a roller (4°C). Binding was followed by washing, elution, and de-crosslinking as described. DNA fragments were precipitated by phenol/chloroform extraction and DNA cleaning with Monarch PCR & DNA Cleanup Kit (NEB) according to the vendor’s instructions. Finally, a size selection was done with Agencourt AMPure XP beads (Beckman Coulter) using 0.55 beads to sample ratio for the selection of fragments below 700 bp, determined empirically.

### ChIP-Seq and data analysis

Fragments were sequenced with a NovaSeq 6000 (100SR, Illumina) in the QB3, and quality control of fragments was done with FastQC (V 0.11.09). Fragments were mapped to the genome with bowtie2 (V 2.5.2), and BAM files were transformed to SAM files with SAMtools (V 1.8). Peak calling was done with MACS2 (V 2.2.9.1), and peaks were visualized on the Integrated Genome Browser (V 9.1.10). Peak sequences that were within 4 kb of a translation start site were used in the MEME suite (V 5.5.5) (https://meme-suite.org/meme/) using the XSTREME option with sequences 4 kb upstream of the translation start sites of all genes as controls. The designated motif size range was between 6 and 21 bp with an E-value ≤0.05. Results were ranked based on the enrichment *P*-value of *ab initio* motifs discovered by the STREME and MEME algorithms by the MEME suite’s SEA algorithm. XSTREME motif *E*-values are estimated for the motif using either the Fisher exact test or the Binomial test while MEME motif *E*-values are estimated using the log-likelihood ratio and based on a different null model than STREME and SEA *E*-values. Therefore, the *E*-values cannot be compared directly. XSTREME motif *E*-values in our analysis do not represent the direct statistical significance, due to the low number of input sequences (81, 82).

### RT-qPCR

RNA was extracted as described above. RNA concentration, quality, and absence of genomic DNA were assessed with Nanodrop and agarose gels. Reverse transcription of 250 ng total RNA was performed with ProtoScript II First Strand cDNA Synthesis Kit (NEB) using anchored oligo dT primers as per the vendor’s instructions. Gene expression was analyzed in technical triplicate by quantitative real-time PCR on a CFX Connect qPCR machine (Bio-Rad) using 2× SYBR select Mastermix (Thermo Fisher Scientific) as per vendor’s instructions and normalized using actin as a housekeeping gene.

### Measurement of enzyme activity

To measure enzyme activity, 10^6^ spores/mL were grown in biological triplicate in 100 mL of VMM medium for 14 hours at 37°C, 250 rpm. Then the culture was filtered on miracloth, washed twice with 50 mL of ddH_2_O, and transferred into 50 mL of VMM with 2% Avicel (Sigma) for 7 days at 37°C, 250 rpm. Endo-1,4-β-D-glucanase (cellulase) activity was measured using Azo-CM-Cellulose dye (Megazyme) according to the vendor’s instructions. The total and secreted protein concentration was measured using Protein Assay Dye (Bio-Rad) and Protein Assay Standard II (Bio-Rad) as per the vendor’s instructions. Enzyme activity was normalized using the total protein concentration.

### Growth curves and conidia quantification

For growth curves, biological triplicates of 400 mL MC medium per strain were inoculated with 10^6^ conidia/mL in a 1 L Erlenmeyer flask, and 100 mL of culture was harvested at the indicated timepoints on #1 Whatman filter paper, dried, and weighed. To quantify conidiation on agar plates, 7-day-old VMM plates in biological quadruplicates per strain were washed with 50 mL ddH2O, filtered through miracloth, and the conidia were counted with a hemocytometer. For quantification of conidia in submerged cultures, 5-day-old cultures on a VMM plate were harvested and 10^6^ conidia were inoculated in 150 mL of CM medium, in biological quadruplicates per strain, and incubated for 10 hours at 37˚C, 250 rpm. 100 mL of the suspension was then transferred on a miracloth filter and washed with ddH_2_O. The mycelia were then transferred to 100 mL PCM or VMM and incubated for 72 h at 37°C, 250 rpm. The culture was then filtered on miracloth and the conidia in the flow through were counted with a hemocytometer.

### Microscopy

Phenotypic analysis of conidiation and strains was performed on an Axioscope2 (Zeiss). Images were captured with a Retiga 2000R camera (QImaging) and iVision (V 4.5.6) software. Image coloration and contrast adjustments were performed with FIJI (V 2.3.0) software.

### Statistical analysis

GraphPad Prism software (V 10.1.1, GraphPad Software Inc.) was used for statistical analyses. For comparing three or more groups, a one-way ANOVA test followed by Tukey’s post hoc test, when applicable, was used. Groups of three or more affected by two variables were analyzed by a two-way ANOVA, followed by a Tukey’s post-test. Results are expressed as the mean ± SD, and a *P* ≤ 0.05 was considered significant.

## Data Availability

*T. thermophilus* strains constructed in this study have been deposited at the Fungal Genetics Stock Center (https://www.fgsc.net/). FGSC numbers for constructed strains are noted in Materials and Methods. GSE273452, GSE273453, and GSE273454 series records are as follows: ChIP-Seq data with wild-type *T. thermophilus*, ∆*res1* P*res1*::*res1*-*V5,* and ∆*res1* P*H2A*::*res1-V5* strains grown for 16 h, https://www.ncbi.nlm.nih.gov/geo/query/acc.cgi?acc=GSE273452; RNA-Seq time course experiment with wild-type *T. thermophilus*, ∆*res1,* and ∆*res1* P*res1*::*res1-gfp* strains from 11.5 to 48 h, https://www.ncbi.nlm.nih.gov/geo/query/acc.cgi?acc=GSE273453; RNA-seq time course with wild-type *T. thermophilus*, ∆*res1,* and ∆*res1* P*H2A*::*res1-V5* strains grown for 6, 7.5, and 9 h, https://www.ncbi.nlm.nih.gov/geo/query/acc.cgi?acc=GSE273454.

## SUPPLEMENTAL MATERIAL

Data set S1 mbio.03111-24-s0001.xlsx Annotation information of the *Thermothelomyces thermophilus* genome.

Data set S2 mbio.03111-24-s0002.xlsx Differentially regulated genes during time course of the WT strain grown in submerged cultures.

Data set S3 mbio.03111-24-s0003.xls GO analsis of six clusters of differentially regulated genes during WT time course.

Data set S4 mbio.03111-24-s0004.xlsx RNAseq analysis of WT versus the res1 mutant and the res1 over-expression strain versus the *res1* mutant.

Data set S5 mbio.03111-24-s0005.xlsx Differentially regulated genes between WT and the *res1* mutant over the 48 h time course.

Data set S6 mbio.03111-24-s0006.xlsx ShinyGO analysis of differentially regulated genes between WT and the *res1* mutant over the time course.

Data set S7 mbio.03111-24-s0007.xlsx Res1 target genes determined by ChIP-seq.

Data set S8 mbio.03111-24-s0008.xlsx Oligonucleotides used in the study.

Supplemental material mbio.03111-24-s0009.pdf Legends for Data sets S1-S8; Tables S1 and S2; Figures S1-S9.

ASM does not own the copyrights to Supplemental Material that may be linked to, or accessed through, an article. The authors have granted ASM a non-exclusive, world-wide license to publish the Supplemental Material files. Please contact the corresponding author directly for reuse.
