# Supplementary material for: Transcriptomic and genetic analysis reveals a Zn2Cys6 transcription factor specifically required for conidiation in submerged cultures of Thermothelomyces thermophilus
Source: mBio. 2024 Nov 27;16(1):e03111-24. doi: 10.1128/mbio.03111-24 (PMC11708020; doi:10.1128/mbio.03111-24)
Supplement: Supplemental material — Legends for Data sets S1-S8; Tables S1 and S2; Figures S1-S9. [file mbio.03111-24-s0009.pdf]

Transcriptomic and genetic analysis reveals a Zn2Cys6 transcription factor specifically required for conidiation in submerged cultures of *Thermothelomyces thermophilus*

Florian Drescher, Yang Li, Jose Manuel, Villalobos-Escobedo, Stefan Haefner, Lori B. Huberman and N. Louise Glass

Supplemental Tables and Figures

Dataset Legends

Dataset S1
Annotation of the *Thermothelomyces thermophilus* genome.
Sheet 1: Re-annotation of the *Thermothelomyces thermophilus* genome.
Sheet 2: Predicted transcription factors in *the T. thermophilus* genome.

Dataset S2
Differentially regulated genes identified during a 48h time course of growth in submerged cultures of wild type *T. thermophilus*.
Sheet 1: WT 14 h versus WT 11.5 h
Sheet 2: WT 16 h versus WT 11.5 h
Sheet 3: WT 20 h versus WT 11.5 h
Sheet 4: WT 48 h versus WT 11.5 h

Dataset S3
Gene ontology analyses of six expression clusters shown in Figure 1.
Sheet 1: Cluster 1
Sheet 2: Cluster 2
Sheet 3: Cluster 3
Sheet 4: Cluster 4
Sheet 5: Cluster 5
Sheet 6: Cluster 6

Dataset S4
RNA-seq Datasets
Sheet 1: RNA-seq data on WT versus the *Δres1* mutant
Sheet 2: RNA-seq data on the *res1* over-expression strain versus the *Δres1* mutant

Dataset S5
Differentially regulated genes in the WT relative to the *Δres1* mutant over a 48 h time course of growth in submerged cultures.
Sheet 1: WT versus the *Δres1* mutant at 11.5 h

Sheet 2: WT versus the *Δres1* mutant at 14 h
Sheet 3: WT versus the *Δres1* mutant at 16 h
Sheet 4: WT versus the *Δres1* mutant at 20 h
Sheet 5: WT versus the *Δres1* mutant at 48 h

Dataset S6
ShinyGO analyses of genes upregulated and downregulat in WT versus the *Δres1* mutant over the 48 h time course of growth in submerged cultures.
Sheet 1: Upregulated genes WT versus the *Δres1* mutant at 11.5 h
Sheet 2: Downregulated genes WT versus the *Δres1* mutant at 11.5 h
Sheet 3: Upregulated genes WT versus the *Δres1* mutant at 14 h
Sheet 4: Downregulated genes WT versus the *Δres1* mutant at 14 h
Sheet 5: Upregulated genes WT versus the *Δres1* mutant at 16 h
Sheet 6: Downregulated genes WT versus the *Δres1* mutant at 16 h
Sheet 7: Upregulated genes WT versus the *Δres1* mutant at 20 h
Sheet 8: Downregulated genes WT versus the *Δres1* mutant at 20 h
Sheet 9: Upregulated genes WT versus the *Δres1* mutant at 48 h
Sheet 10: Downregulated genes WT versus the *Δres1* mutant at 48 h

Dataset S7
Res1 target genes determined by chromatin-immunoprecipation-sequencing.
Sheet 1: ChIP peaks
Sheet 2: Res1 binding sites determined by SXTREME analyses.

Dataset S8
Oligonucleotides used in the study.
Sheet 1: Oligonucleotides used for mutant construction.
Sheet 2: Oligonucleotides used for plasmid construction.
Sheet 3: Oligonucleotides used for RT-qPCR

Table S1. Genes involved in conidiation in *N. crassa* and *A. nidulans* that are conserved in *T. thermophilus*

Gene name	Organism	Gene ID	*T. thermophilus homolog*	Query cover	E-value	Percent Identity
ada-6	*N. crassa*	NCU04866	MYCTH_2305551	83%	0	59.38%
gna-3	*N. crassa*	NCU05206	MYCTH_2309848	99%	0	85.39%
gnb-1	*N. crassa*	NCU00440	MYCTH_2304670	99%	0	92.96%
fluffy/fl	*N. crassa*	NCU08726	MYCTH_2071661	94%	0	42.68%
con-6	*N. crassa*	NCU08769	MYCTH_2315464	93%	3.00e-23	50.00%
con-8	*N. crassa*	NCU09235	MYCTH_2296064	31%	6.00e-18	67.86%
con-10	*N. crassa*	NCU07325	MYCTH_2310279	98%	9.00e-42	80.00%
acon-2	*N. crassa*	NCU00478	MYCTH_2304708	98%	0	39.83%
acon-3	*N. crassa*	NCU07617	MYCTH_2305657	97%	0	52.59%
csp-1	*N. crassa*	NCU02713	MYCTH_2313746	70%	7.00e-58	47.60%
csp-2	*N. crassa*	NCU06095	MYCTH_2082522	100%	0	65.91%
asm-1	*N. crassa*	NCU01414	MYCTH_93313	98%	0	57.71%
stuA	*A. nidulans*	ANIA_05836	MYCTH_93313	89%	2.00e-119	45.56%
flb-3	*N. crassa*	NCU03043	MYCTH_2297037	98%	1.00e-155	64.25%

Table S2. Predicted transcription factors in *T. thermophilus* with increased expression levels early in the conidiation process

Cluster	Gene	PFAM domains	PFAM/InterPro description	*N. crassa* homolog	*N. crassa* gene name	E-value	% Identity
2	MYCTH_2294400	PF02178	AT hook motif	NCU02068	hypothetical protein	3e-78	50.00%
	MYCTH_2135800	PF00170; PF07716	bZIP transcription factor	NCU08055	*zip-1*	4e-21	56.58%
	MYCTH_2301118	PF00046	homeobox domain	NCU09556	*sgr-29*	0.0	59.72%
	MYCTH_46530	PF05224	NDT80 / PhoG like DNA-binding family	NCU03725	*vib-1*	0.0	67.69%
	MYCTH_2300984	PF00172	Fungal Zn(2)-Cys(6) binuclear cluster domain	NCU01386	*ada-10*	6e-114	49.18%
	MYCTH_2312847	PF00172; PF04082	Fungal Zn(2)-Cys(6) binuclear cluster domain	NCU06799	*vad-5*	0.0	54.19%
	MYCTH_2298236	PF04769	Mating-type protein MAT alpha 1	NCU01958	*mat A-1*	4e-48	43.55%
	MYCTH_2304956	PF00172	Fungal Zn(2)-Cys(6) binuclear cluster domain	NCU00282	*tah-5*	3e-100	31.11%
	MYCTH_64004	NA	Fungal Zn(2)-Cys(6) binuclear cluster domain	NCU08527	hypothetical protein	0.001	29.69%
5	MYCTH_102871	PF00172; PF04082	Fungal Zn(2)-Cys(6) binuclear cluster domain	NCU07705	*clr-1*	4e-56	28.55%
	MYCTH_109046	PF00249	Myb-like DNA-binding domain	NCU01706	*vsd-6*	1e-106	59.32%
	MYCTH_2060942	PF00172; PF04082	Fungal Zn(2)-Cys(6) binuclear cluster domain	NCU03110	hypothetical protein	3e-47	31.63%
	MYCTH_2061546	NA	CisBP description Myb/SANT	NCU05681	hypothetical protein	3e-115	51.20%
	MYCTH_2066181	PF00172; PF04082	Fungal Zn(2)-Cys(6) binuclear cluster domain	NCU10080	hypothetical protein	0.0	56.86%
	MYCTH_2121737	PF00172	Fungal Zn(2)-Cys(6) binuclear cluster domain	NCU05414	hypothetical protein	8e-143	51.72%
	MYCTH_2293968	PF10680	RNA polymerase I specific transcription initiation factor	NCU00926	hypothetical protein	2e-112	39.94%

	MYCTH_2295721	PF00172	Fungal Zn(2)-Cys(6) binuclear cluster domain	NCU02525	hypothetical protein	1e-160	48.13%
	MYCTH_2295908	PF00172; PF11951	Fungal Zn(2)-Cys(6) binuclear cluster domain	NCU07392	*adv-1*	0.0	73.87%
	MYCTH_2296180	PF00505; PF09011	HMG (high mobility group) box; HMG-box domain	NCU03126	hypothetical protein	e-132	62.25%
	MYCTH_2296328	PF00046	homeobox domain	NCU03070	hypothetical protein	7e-131	42.76%
	MYCTH_2297012	PF00170; PF03131; PF07716	bZIP transcription factor	NCU00499	*ada-1*	0.0	65.98%
	MYCTH_2297445	NA IPR001138	Fungal Zn(2)-Cys(6) binuclear cluster domain	NCU00558	hypothetical protein	5e-115	38.21%
	MYCTH_2298994	PF00172; PF04082	Fungal Zn(2)-Cys(6) binuclear cluster domain	NCU08294	*nit-4*	0.0	76.12%
	MYCTH_2300332	PF00250; PF00498	Forkhead domain	NCU11138	hypothetical protein	0.0	65.53%
	MYCTH_2300935	PF00172; PF04082	Fungal Zn(2)-Cys(6) binuclear cluster domain	NCU03643	*far-2*	0.0	42.17%
	MYCTH_2302052	NA IPR013087	Zinc finger C2H2-type	NCU03699	*res-1/znf-13*	0.0	67.43%
	MYCTH_2303722	PF00172; PF04082	Fungal Zn(2)-Cys(6) binuclear cluster domain	NCU06407	*vad-3*	0.0	67.32%
	MYCTH_2304475	PF00250; PF00498	Forkhead domain	NCU00019	*fkh-1*	0.0	65.62%
	MYCTH_2304982	PF00172	Fungal Zn(2)-Cys(6) binuclear cluster domain	NCU00289	*tah-1*	0.0	68.34%
	MYCTH_2305582	PF00172	Fungal Zn(2)-Cys(6) binuclear cluster domain	NCU02525	*sgr-4*	1e-04	29.37%
	MYCTH_2309330	PF00320; PF00989; PF08447; PF13426	GATA zinc finger; PAS-domain	NCU02356	*wc-1*	0.0	51.46%
	MYCTH_2309668	PF00172	Fungal Zn(2)-Cys(6) binuclear cluster domain	NCU10597	*fgr-1*	1e-47	30.37%
	MYCTH_2310172	PF00172; PF04082	Fungal Zn(2)-Cys(6) binuclear cluster domain	NCU06990	hypothetical protein	0.0	62.97%

	MYCTH_2311492	PF00172; PF04082	Fungal Zn(2)-Cys(6) binuclear cluster domain	NCU03489	*sah-4*	0.0	45.31%
	MYCTH_2311613	PF00172; PF04082	Fungal Zn(2)-Cys(6) binuclear cluster domain	NCU03417	hypothetical protein	0.0	62.80%
	MYCTH_53224	PF00172; PF04082	Fungal Zn(2)-Cys(6) binuclear cluster domain	NCU09033	*pdr-1*	0.0	59.06%
	MYCTH_59388	PF04855	SNF5 / SMARCB1 /INI1<br>CisBP: GATA factor	NCU07893	*pdb-5*	0.0	70.89%
	MYCTH_71014	PF00096	Zinc finger, C2H2 type	NCU06145	*ada-21*	1e-133	63.38%
	MYCTH_73708	PF00172; PF11951	Fungal Zn(2)-Cys(6) binuclear cluster domain	NCU05294	*znf-40*	0.0	55.01%

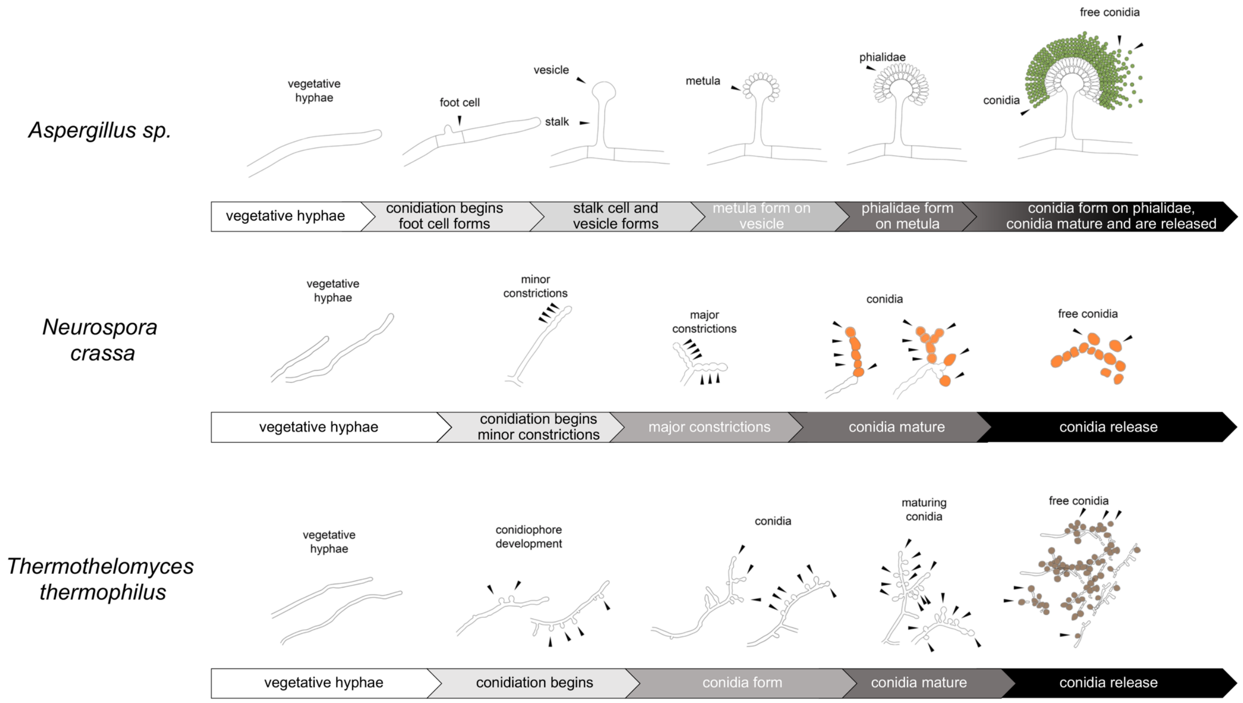

Figure S1. Comparison between developmental aspects of the conidiation process between *Aspergillus sp.*, *Neurospora crassa*, and *Thermothelomyces thermophilus*. From (1, 2) and this work. In Aspergilli, conidia are formed by budding from a conidigenious cell that expands from an inner cell wall layer to form a conidium. A new conidium pushes the older one out, creating the characteristic brush structure of Aspergillus species. In Thermothelomyces, blastoconidia are derived by budding from a conidiogenous cell before separation by a septum. In Neurospora, the conidium forms by elongation of an aerial hypha rather than budding from a conidigenious cell, which subsequently gets partitioned by septa into individual conidia (3).

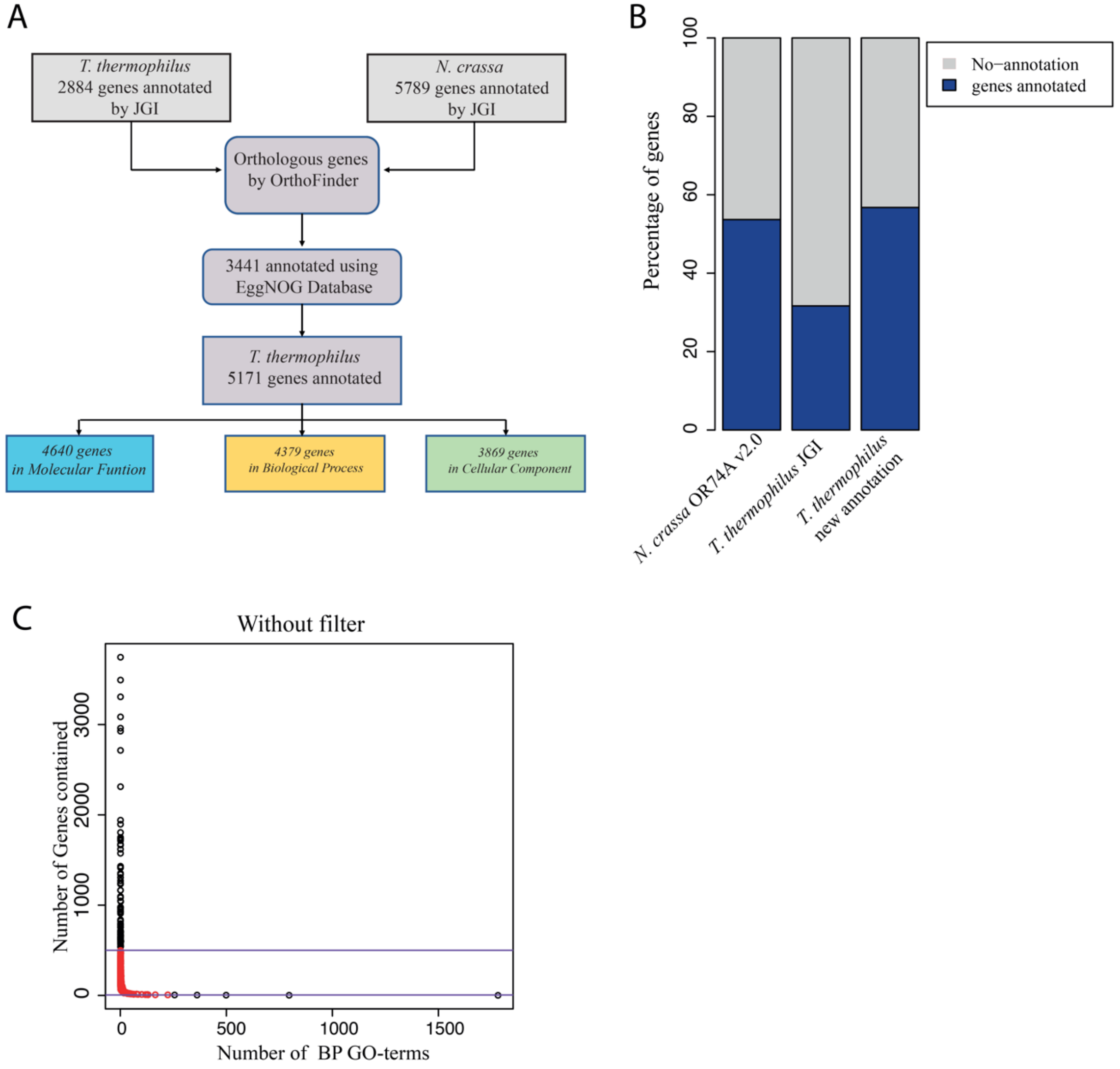

Fig. S2. A: Schematic workflow of annotating the *T. thermophilus* genome. First OrthoFinder (4) was used to find orthologs of *T. thermophilus* genes in the annotated *N. crassa* genome. In a second step additional genes were annotated with the EggNOG database (5), resulting in a total of 5171 annotated genes (Dataset S1). B: bar graphs visualizing the increase of annotated genes in the *T. thermophilus* genome from 31.7% (2884/9097 genes) to 56.8% (5171/9097 genes). C: Distribution of GO terms for functional category enrichment without applying a filter shows many GO terms have a low number (under 5) of genes per term and some genes have a high number of GO terms (over 500) ascribed to them (both indicated in black).

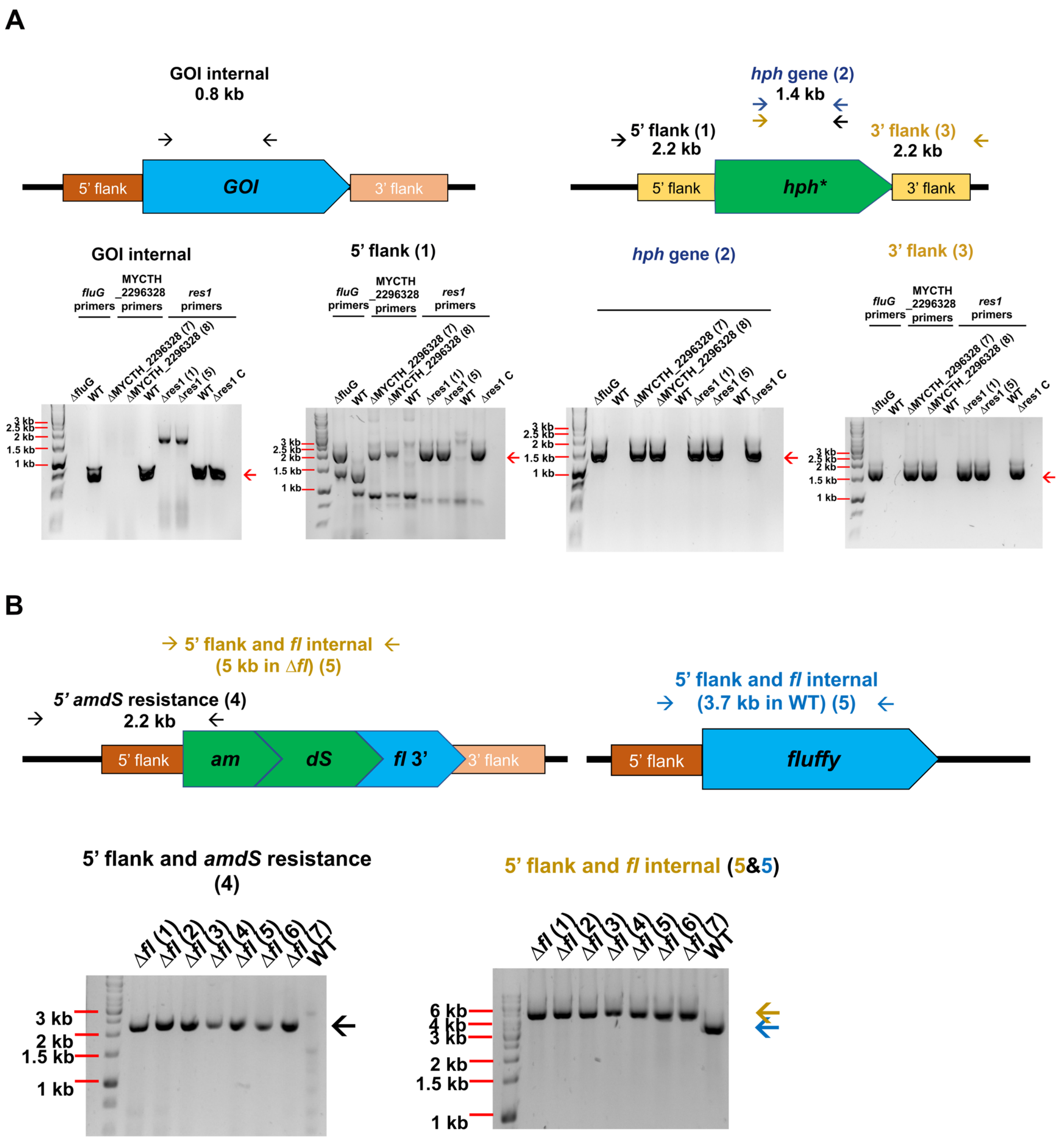

Fig. S3. A: Top Left: Schematic of the PCR oligonucleotide binding sites used for the different PCRs to verify the presence of deletions. Position of oligonucleotides used for identification by PCR that the gene of interest (GOI) was deleted. No amplification is expected for successful deletions. Top Right: The detection of successful integration of the marker (*hph*) in deletion strain used oligonucleotides in the 5' flank (black) and an oligonucleotide binding site in the *hph* marker gene (black) and an oligonucleotide in the 3' flank (yellow) with an internal *hph* oligonucleotide (yellow). Finally, two primers within the *hph* gene were used to assess integration of the *hph* cassette. No amplification with the black pair, yellow pair or blue pair is expected in the WT. Bottom: Agarose gel pictures of PCRs for the GOI that was deleted, 5' flank (oligonucleotide pair 1, black), *hph/hyg*^{R} resistance cassette (oligonucleotide pair 2, blue) and 3' flank

(oligonucleotide pair 3, yellow). Top Left: Schematic of oligonucleotide binding sites for the verification of the *fl* deletion strain using an *amdS* split marker (6, 7). The 5’ part of the *amdS* gene was fused with 1 kbp of the 5’ flanking region of *fl* with an overlap with the 3’ part of the *amdS* split marker. The 3’ *amdS* part was fused with 1.2 kbp of the *fl* 3’ flank. The correct integration was tested with oligonucleotide pair 4 (black) binding outside of the 5’ flank of the construct and inside of the *amdS* gene. An amplification indicates correct genomic integration of the construct. Oligonucleotide pair 5 binds in the 5’ flank and in the 3’ part of *fl*. In the *fl* deletion strain, the amplicon using the yellow primers contain the *amdS* marker, which is 5 kbp in size. In the WT (blue) the *amdS* marker is missing and the amplicon is 3.7 kbp in length. Bottom: Agarose gel pictures of PCRs, binding outside of the 5’ flank and inside the *amdS* marker gene and inside the 5’ flank (left picture, oligonucleotide pair 4). Right picture (oligonucleotide pair 5) binding of oligonucleotides in the 5’ flank and the 3’ end of the *fl* gene that was fused with the 3’ split marker (right). The *amdS* split marker is missing in the WT, resulting in an expected amplicon size of 3.7 kb (blue arrow) and 5 kbp in the deletion strains (yellow arrow) as the *amdS* marker is integrated. See Dataset S8 for oligonucleotide sequences.

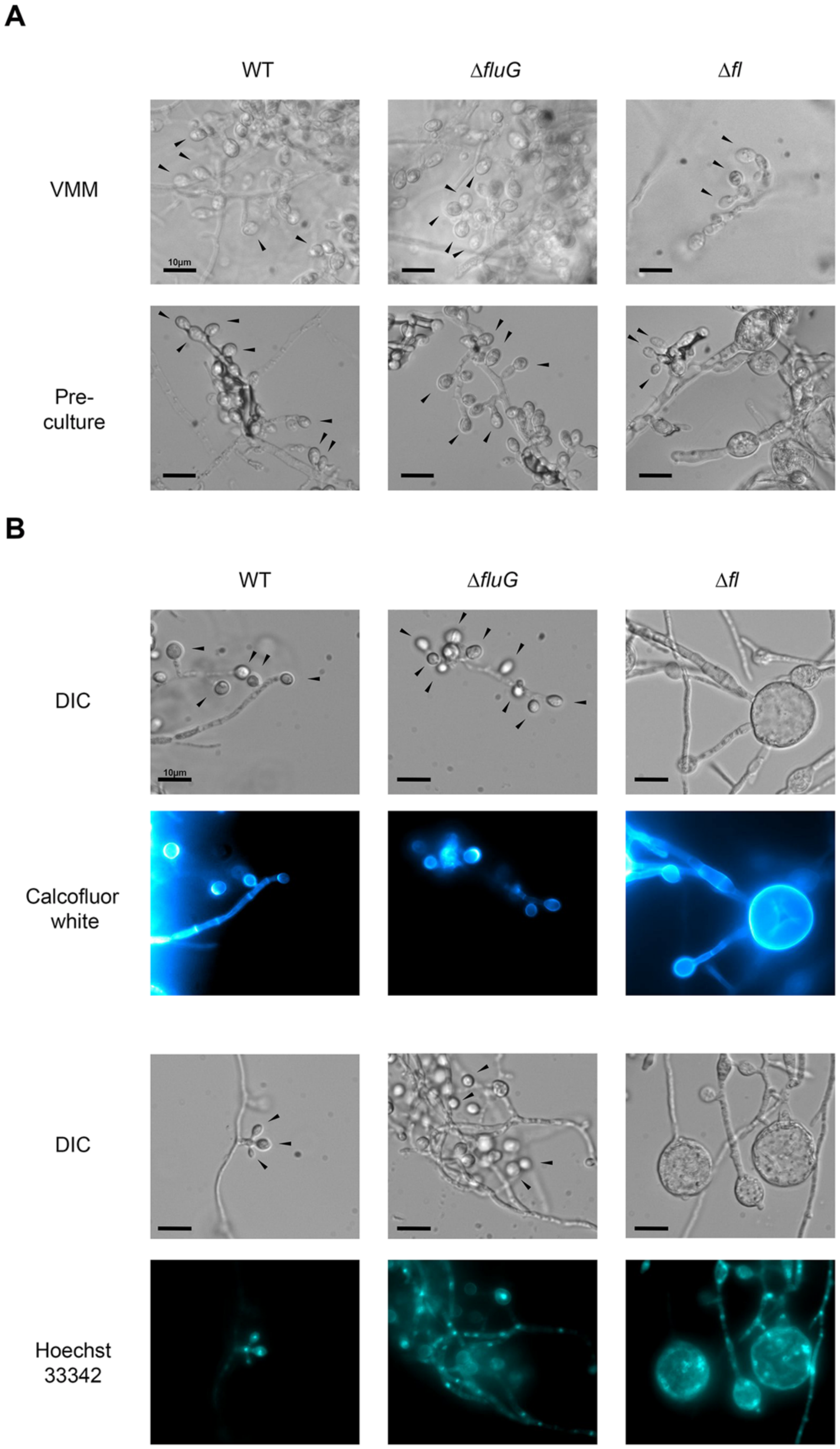

Figure S4. A: DIC microscopic images of *T. thermophilus* WT, *ΔfluG*, and *Δfl* growing on plates after 5 days of growth. Picture taken at the edge of the colony on either VMM (8) or PCM medium, as indicated (9). Black arrows indicate conidia. B: Microscopic images of submerged cultures of *T. thermophilus* WT, *ΔfluG*, and *Δfl* strains after 48 h of incubation in PCM. Cell wall was stained with calcofluor white (1 µg/mL) and nuclei were stained with Hoechst 33342 (1 µg/mL). Black arrows indicate conidia.

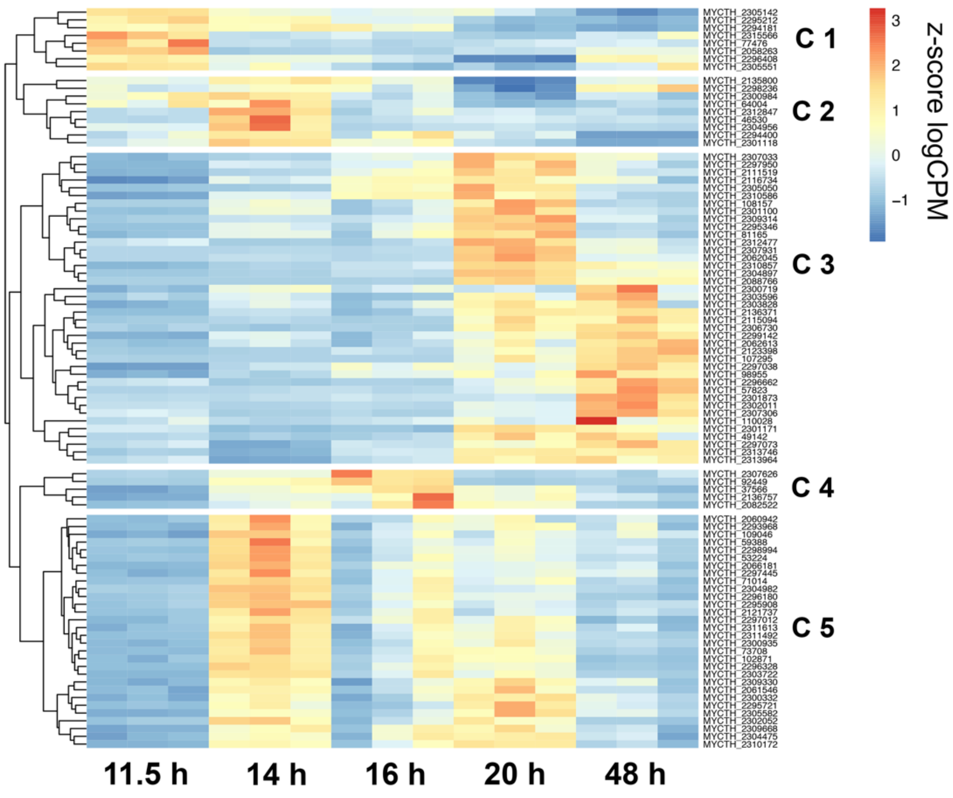

Fig. S5. Heatmap of hierarchical clustering of transcription factor genes differentially expressed at any time point as compared to expression at 11.5 h (92 of 264 total predicted TFs) (Dataset S1) during the time course of WT growth in submerged cultures in PCM. Shown are triplicates normalized with the z-scores of the logCPM expression. Clusters are numbered C1 to C5.

A

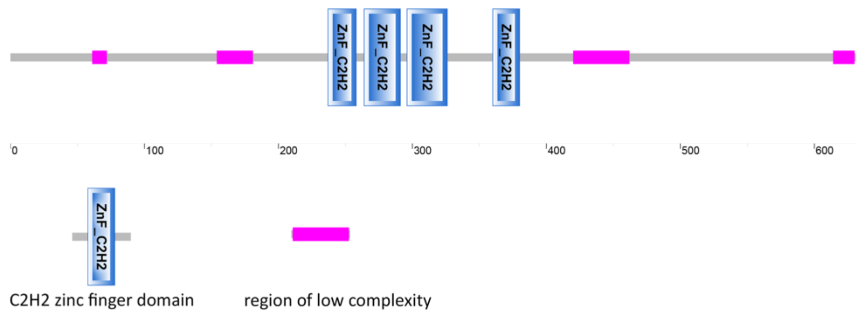

B

```
MYCTH_2302052   ----------------------------MDLGLSPEPYSGAGYSRTAPGFPTTMAYEN-G 31
NCU03699        MATARHYEADEYTMHYPEPPQGGMFSTPVDMGISPEYYPPR-----SQEYTTSMAYDQST 55
                                            :*:*:*** *        :  : *:***::

MYCTH_2302052   AVYAEAPYAYASGRASPGMYADDGDVVRGPASDMSMSAPSSNMGSPLSSHGQAAPIPDWA 91
NCU03699        TLYADAPYNMYHA-KSPSLYPEDSNLSVD-----SHSASSSNAGSPLSNPGNL---ASW- 105
                ::**:***    .  **.:* :*.::  .     * ** *** *****. *:     .*

MYCTH_2302052   SAPHGLGLTPGIVDQGDYLPGGEYSFAPGGIESFNQPSFDFPAAKAPGFVDPSLIHPDVR 151
NCU03699        GAPLGPGVSPGIVDQGEFFHGNEYSFTPLDG---YNPNFEFSVPKGPGFVDPSLIHPEVR 162
                .** * *::*******::: *.****:* .     :*.*:* . *.***********:**

MYCTH_2302052   PMAMPYEPSYPAPPSSGYPASPSLSVSASPQLRPGSSSPFMHNGNYQPYSPFQPPVDPQR 211
NCU03699        SMAMPSYEQMYPAPNSAYPGSPAL--SASPQLRTGSTSPFLQNNNYPAYSPFPAPLEPQR 220
                 ****   .    *.*.**.**:*  ******* **:***::*.**  ****  *::***

MYCTH_2302052   RGSLVSYQSHFSGEQPFGGDESRDKQRCPHQDCGKTFKDLKAHMLTHQNERPEKCPITTC 271
NCU03699        RQSLVSYQSNLSEQPYSGDETLKEKQRCPHPECGKTFKDLKAHMLTHQTERPEKCPIATC 280
                * *******::* :   *.:  ::****** :****************.********:**

MYCTH_2302052   EYHVKGFARKYDKNRHTLTHYKGTMVCGFCPGSGSAAEKSFNRADVFKRHLTAVHGVEQT 331
NCU03699        EYHVKGFARKYDKNRHTLTHYKGTMVCGFCPGSGSAAEKSFNRADVFKRHLTAVHGVEQT 340
                ************************************************************

MYCTH_2302052   PPNGRKKTAGGSS-SGNDAKLTGYAPDATGKCSTCSQTFSNAQDFYEHLDDCVLRIVQQE 390
NCU03699        PPNARKKTGGVNGGNSGNKKLTGYAPDATGKCSTCSQTFSNAQDFYEHLDDCVLRIVQQE 400
                ***.****.* .. ...: *****************************************

MYCTH_2302052   DPAEAINAQRLAEVENDQEVHETLEKNHLPTTTTTTTPTTTNTTITTTMTSTASDAADED 450
NCU03699        DPTEAINAKRLAEVENDLDVHNTLEKNNLPTTTTMTTQM---------------DDIDED 445
                **:*****:******** :**:*****:****** **                 *  ***

MYCTH_2302052   DENMEDDDEDDELKGGRRGAAPKGTLCSPRKAKVGGGNPPNGVQKSRGMTHSRGGVPLHT 510
NCU03699        -EDMVDEDDNEDPVGGRGS------KSS--PSRKTKGNPANGVQKSRGLTHSRGGVALQS 496
                 *:* *:*::::  *** .       .*   ::   *** ********:******* *::

MYCTH_2302052   RARGRKNRRDYPSSWGFDKGQMTMKKRVLAVFDGPRRLAKDDMMLSTEQEVRIKLQDGKS 570
NCU03699        KARGRKNRRDYPSSWGFDKGQMTMKKRVVAVFDGPRRLAKDDMMLSTEHEVRIKLQDGKS 556
                :***************************:*******************:***********

MYCTH_2302052   YVTDLDLQTLKRAEGFLNATEEEKGPWISDDPTEEQFKQMLEFSAAAAASSSEAPPAAAA 630
NCU03699        YVTDLDVQTLRRTEGFLNATEEEKGPWISDDPTEEEKEEARRLLMLQ------------- 603
                ******:***:*:**********************: ::  .:

MYCTH_2302052   Q       631
NCU03699        -       603
```

Figure S6. A: Cartoon of Res1 domains showing C2H2 zinc finger domains and low complexity regions as predicted by SMART (10). B: Amino acid alignment of *T. thermophilus* Res1 (MYCTH_2302052) and *N. crassa* RES-1 (NCU03699) using CLUSTAL O (V 1.2.4). Highlighted with red squares are the four predicted zinc finger domains (Cis-BP). The two TFs have an overall amino acid identity of 73.9%. The four C2H2 core regions (highlighted with red boxes) show a 97% amino acid sequence identity. Identical residues are marked by an asterisk (*****). Conservation between amino acids of strongly similar properties (> 0.5 Gonnet PAM 250 matrix) are marked by colon (**:**) and conservation between amino acids with weakly similar properties (≤0.5 and > 0 Gonnet PAM 250 matrix) are marked by period (**.**).

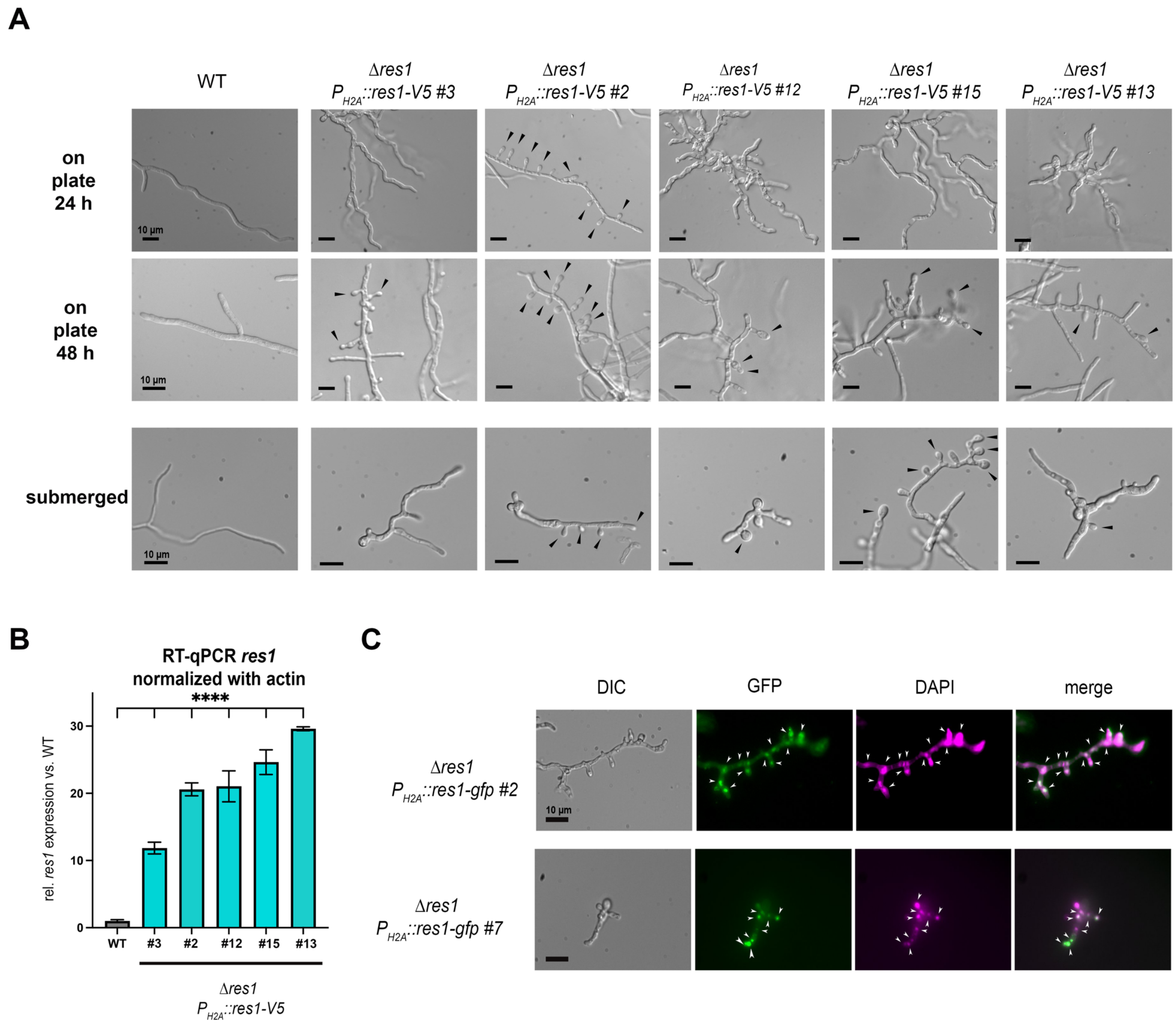

Figure S7. A: Microscopic images of *T. thermophilus* WT and *Δres1 P_{H2A}::res1-V5* strains on agar plates, showing the edge of a 24 h and 48 h old colony and in submerged culture after 12 h of growth in PCM (9). Scale bar 10 µm, black arrows indicate conidiophores/conidia. B: RT-qPCR of *T. thermophilus res1* mRNA expression levels of cultures harvested after 12 h comparing WT *res1* expression and the indicated *Δres1 P_{H2A}::res1-V5* strains. Expression normalized to actin and relative to *res1* expression in the WT strain. One-way ANOVA with a Tukey's post-hoc test, comparing the *Δres1 P_{H2A}::res1-V5* strains with the WT **** $p \leq 0.0001$. C: Fluorescence microscopy images of *res1* in *P_{H2A}::res1-gfp* strains #2 and #7. Controls are the same as in Figure 5 of the main text. Scale bar 10 µm, white arrows indicate nuclei.

A

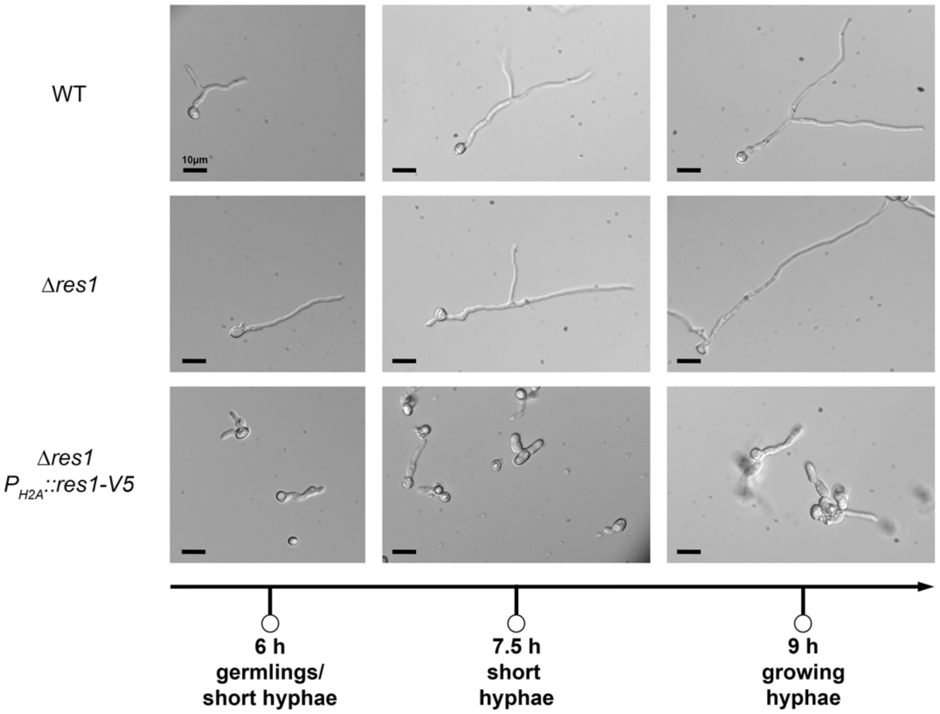

B

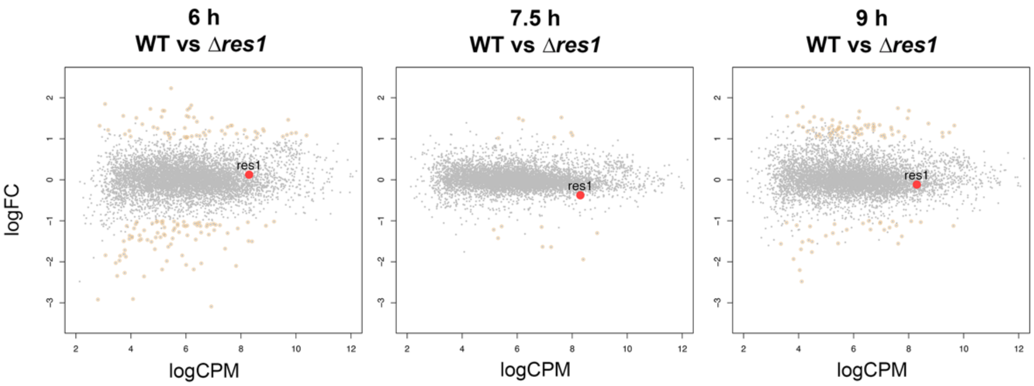

Fig. S8. A: Microscopic images of *T. thermophilus* WT, Δ*res1*, and Δ*res1* P_{H2A}::*res1-V5* strains at 6, 7.5, and 9 h after inoculation in PCM (9) and which were used for RNAseq analysis. Scale bar 10 µm. B: Dot plots of differentially regulated genes between WT and Δ*res1* strains at the marked timepoints. Expressed genes in grey, differentially regulated genes in peach, *res1* in red. Number of differentially regulated genes between the WT strain and the Δ*res1* mutant at 6 h (99 genes); 7.5h (15 genes); 9h (145 genes).

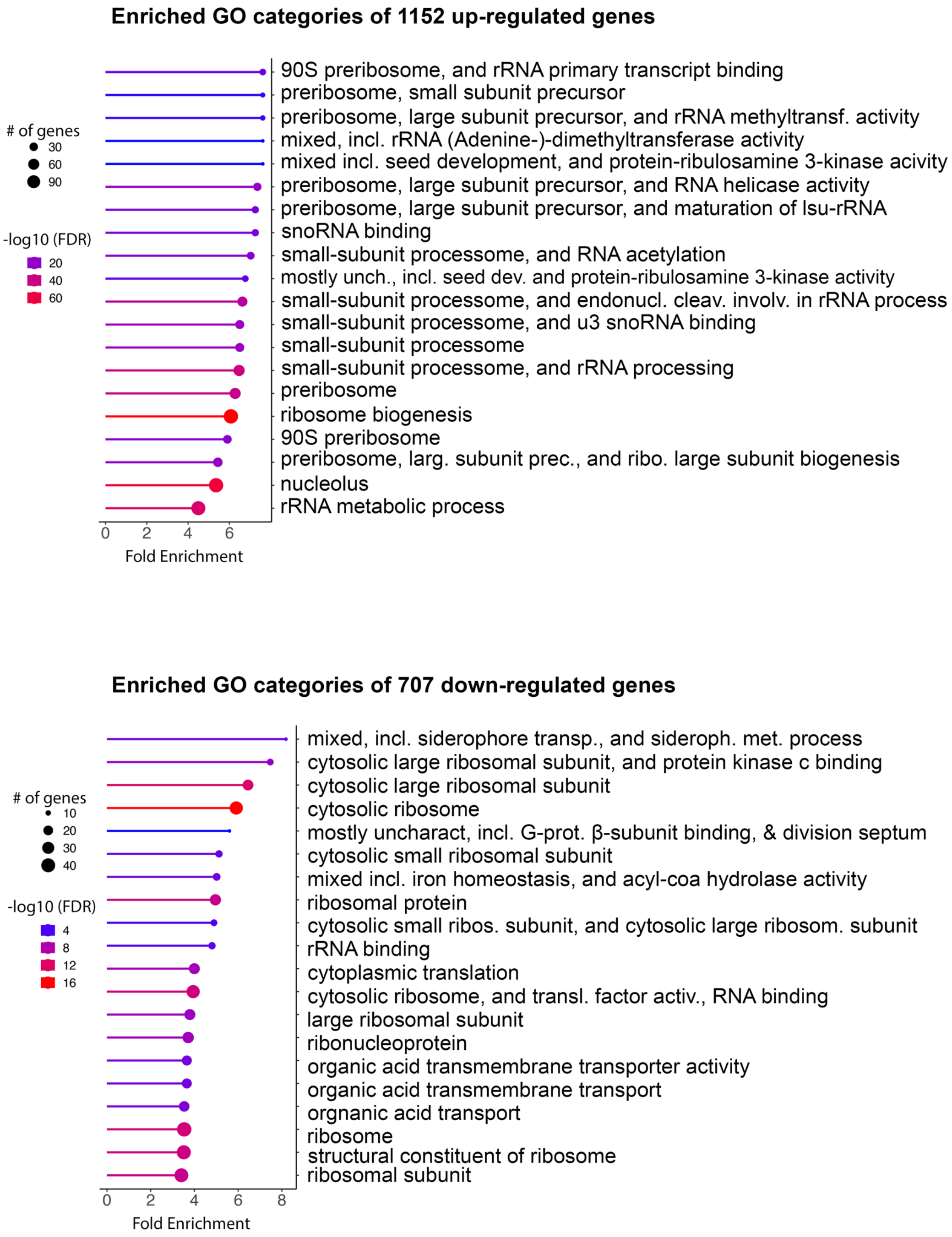

Figure S9. Functional category enrichment of all unique up- and down-regulated genes between *T. thermophilus Δres1 P_{H2A}::res1-V5* and *Δres1* mutant or WT at all time points. Displayed are the fold enrichment, number of genes and FDR in the highest enriched categories with FDR ≤0.05 using ShinyGO (v0.8) (11).

References

1. Adams TH, Wieser JK, Yu JH. 1998. Asexual sporulation in *Aspergillus nidulans*. Microbiol Mol Biol Rev. 62(1):35-54.

2. Springer ML, Yanofsky C. 1989. A morphological and genetic analysis of conidiophore development in *Neurospora crassa*. Genes Dev. 3(4):559-71.
3. Ebbole DJ. 2010. The Conidium. In: Borkovich KA, Ebbole DJ, editors. Cellular and Molecular Biology of Filamentous Fungi: American Society of Microbiologyp. 577-90.
4. Emms DM, Kelly S. 2019. OrthoFinder: phylogenetic orthology inference for comparative genomics. Genome Biol. 20(1):238.
5. Cantalapiedra CP, Hernandez-Plaza A, Letunic I, Bork P, Huerta-Cepas J. 2021. eggNOG-mapper v2: Functional Annotation, Orthology Assignments, and Domain Prediction at the Metagenomic Scale. Mol Biol Evol. 38(12):5825-9.
6. Liu Q, Gao R, Li J, Lin L, Zhao J, Sun W, Tian C. 2017. Development of a genome-editing CRISPR/Cas9 system in thermophilic fungal Myceliophthora species and its application to hyper-cellulase production strain engineering. Biotechnol Biofuels. 10:1.
7. Kwon MJ, Schutze T, Spohner S, Haefner S, Meyer V. 2019. Practical guidance for the implementation of the CRISPR genome editing tool in filamentous fungi. Fungal Biol Biotechnol. 6:15.
8. Vogel HJ. 1956. A convenient growth medium for *Neurospora*. Microbiol Genet Bull. 13:42-6.
9. Haefner S, Thywissen S, Hartmann H, Boehmer N, inventors. 2016. Method of producing proteins in filamentous fungi with decreased clr2 activity. USA patent US10597429B2.
10. Letunic I, Khedkar S, Bork P. 2021. SMART: recent updates, new developments and status in 2020. Nucleic Acids Res. 49(D1):D458-D60.
11. Ge SX, Jung D, Yao R. 2020. ShinyGO: a graphical gene-set enrichment tool for animals and plants. Bioinformatics. 36(8):2628-9.
